# Bacterial Effector Activates Jasmonate Signaling by Directly Targeting JAZ Transcriptional Repressors

**DOI:** 10.1371/journal.ppat.1003715

**Published:** 2013-10-31

**Authors:** Shushu Jiang, Jian Yao, Ka-Wai Ma, Huanbin Zhou, Jikui Song, Sheng Yang He, Wenbo Ma

**Affiliations:** 1 Department of Plant Pathology and Microbiology, University of California, Riverside, California, United States of America; 2 Center for Plant Cell Biology, University of California, Riverside, California, United States of America; 3 DOE Plant Research Laboratory, Michigan State University, East lancing, Michigan, United States of America; 4 Department of Biochemistry, University of California, Riverside, California, United States of America; 5 Howard Hughes Medical Institute, Chevy Chase, Maryland, United States of America; 6 Institute of Integrative Genomics, University of California, Riverside, California, United States of America; University of Nebraska, United States of America

## Abstract

Gram-negative bacterial pathogens deliver a variety of virulence proteins through the type III secretion system (T3SS) directly into the host cytoplasm. These type III secreted effectors (T3SEs) play an essential role in bacterial infection, mainly by targeting host immunity. However, the molecular basis of their functionalities remains largely enigmatic. Here, we show that the *Pseudomonas syringae* T3SE HopZ1a, a member of the widely distributed YopJ effector family, directly interacts with jasmonate ZIM-domain (JAZ) proteins through the conserved Jas domain in plant hosts. JAZs are transcription repressors of jasmonate (JA)-responsive genes and major components of the jasmonate receptor complex. Upon interaction, JAZs can be acetylated by HopZ1a through a putative acetyltransferase activity. Importantly, *P. syringae* producing the wild-type, but not a catalytic mutant of HopZ1a, promotes the degradation of HopZ1-interacting JAZs and activates JA signaling during bacterial infection. Furthermore, HopZ1a could partially rescue the virulence defect of a *P. syringae* mutant that lacks the production of coronatine, a JA-mimicking phytotoxin produced by a few *P. syringae* strains. These results highlight a novel example by which a bacterial effector directly manipulates the core regulators of phytohormone signaling to facilitate infection. The targeting of JAZ repressors by both coronatine toxin and HopZ1 effector suggests that the JA receptor complex is potentially a major hub of host targets for bacterial pathogens.

## Author Summary

Many Gram-negative bacterial pathogens rely on the type III secretion system, which is a specialized protein secretion apparatus, to inject virulence proteins, called effectors, into the host cells. The type III secreted effectors (T3SEs) directly target host substrates in order to promote bacterial colonization and disease development. Therefore, the identification and characterization of the direct host targets of T3SEs provides important insights into virulence strategies employed by bacterial pathogens to cause diseases. Here, we report that the plant pathogen *Pseudomonas syringae* T3SE HopZ1a physically interacts with and modifies the jasmonate ZIM-domain (JAZ) proteins in plant hosts. JAZ proteins are components of the receptor complex of the plant hormone jasmonates (JA) and key transcription repressors regulating JA-responsive genes. HopZ1a belongs to the widely distributed YopJ (for Yersinia Outer Protein J) family of T3SEs with a potential acetyltransferase activity. *P. syringae* producing HopZ1a, but not the catalytic mutant, leads to the degradation of AtJAZ1 during infection. As a result, HopZ1a activates JA signaling and promotes bacterial multiplication in *Arabidopsis*. This work provides the first example of a bacterial effector that subverts host immunity by directly targeting the receptor complex of a defense-associated hormone in plants.

## Introduction

A prevailing concept for plant-pathogen interactions highlights the continuing battles between the activation of plant immune responses upon pathogen perception and the subversion of host immunity by virulence factors produced by successful pathogens. One branch of the plant immunity system is based on the recognition of pathogen- or microbe-associated molecular patterns (PAMP/MAMPs), which leads to a signal transduction cascade, and eventually *PAMP-triggered immunity* (PTI) [1]. PTI, broadly referred as basal defense in plants, restricts the growth of the vast majority of potential pathogens encountered by plants in the surrounding environment [2], [3]. However, successful pathogens produce virulence factors to effectively suppress PTI. For example, Gram-negative bacterial pathogens, such as *Pseudomonas syringae*, inject type III-secreted effectors (T3SEs) into the host cell to actively inhibit PTI [4], [5]. As a counter-attack strategy, plants have evolved nucleotide-binding leucine-rich repeat (NB-LRR) proteins to perceive specific T3SEs, directly or indirectly, and elicit *effector-triggered immunity* (ETI), which is often associated with localized programmed cell death at the infection sites [2], [3], [6].

Recent studies suggest that many *P. syringae* T3SEs suppress PTI and/or ETI by targeting important components of plant immunity [5], [7], [8]. Although the virulence targets of a few T3SEs have been characterized, the molecular mechanisms by which the majority of T3SEs subvert host resistance or facilitate nutrient acquisition remain elusive. HopZ1 is a *P. syringae* T3SE that belongs to the widely distributed YopJ family of cysteine proteases/acetyltransferases produced by both plant and animal bacterial pathogens [9]. The YopJ-like T3SEs share a conserved catalytic core, consisting of three key amino acid residues (histidine, glutamic acid, and cysteine), which is identical to that of clan-CE (C55-family) cysteine proteases [10]. However, several members of the YopJ effector family have been shown to possess acetyltransferase activity. YopJ and VopA modify their target proteins (mitogen-associated protein kinases and Ikkα/β) in animal hosts and the acetylation of these host targets blocks their phosphorylation and the subsequent defense signal transduction [11], [12]. PopP2 produced by the plant pathogen *Ralstonia solanacerum* has an autoacetylation activity, which is essential for its recognition in resistant plants; however, whether PopP2 can modify its target proteins in the host remains unknown [13].

Two functional HopZ1 alleles, HopZ1a and HopZ1b, have been identified in *P. syringae* [9]. HopZ1b is produced by *P. syringae* pv. *glycinea* (*Pgy*) strains, which are the causal agents of bacterial blight disease on soybean (*Glycine max*) [9]. HopZ1b*_Pgy_*_BR1_ (HopZ1b in *Pgy* strain BR1; hereafter HopZ1b) promotes *P. syringae* multiplication in soybean; whereas the closely-related HopZ1a*_Psy_*_A2_ (HopZ1a in *P. syringae* pv. *syringae* strain A2; hereafter HopZ1a) triggers an HR in soybean cultivar Williams 82 and *Arabidopsis thaliana* accession Columbia-0 (Col-0, wild-type) [14]. HopZ1 mutants with the catalytic cysteine residues (C216 in HopZ1a or C212 in HopZ1b) substituted by alanines lose the virulence function or the HR-triggering activity, indicating that the functions of HopZ1 alleles require their enzymatic activities [9], [14]. In addition, HopZ1 has a potential N-terminal myristoylation site (Gly2) which directs the proteins to the plasma membrane [14], [15]. This myristoylation site of HopZ1a contributes to its avirulence function in both soybean and *Arabidopsis* [14], [15]. However, it is not clear whether this myristoylation site is important for the virulence function of HopZ1a. HopZ1 exhibited weak cysteine protease activities in vitro [9]. Recent studies showed that HopZ1a also possessed an acetyltransferase activity and could use tubulin as a substrate in vitro. Modification of tubulin is associated with the disruption of microtubule cytoskeleton, which may contribute to bacterial pathogenesis [16].

To identify potential host targets of HopZ1, we conducted yeast two-hybrid screening using a cDNA library of the natural host soybean and identified several HopZ1-interacting proteins (ZINPs). ZINP1 (2-hydroxyisoflavanone dehydratase, GmHID1) is a key enzyme in the soybean isoflavone biosynthetic pathway and a positive regulator of soybean basal defense. HopZ1 induces the degradation of GmHID1, and hence a decreased isoflavone production in soybean, resulting in increased plant susceptibility to bacterial infection [17]. HopZ1 also enhances bacterial infection in *Arabidopsis*, which does not have a putative ortholog of GmHID1. To understand the mechanisms underlying the virulence function of HopZ1a in *Arabidopsis*, we characterized another family of ZINPs, which were identified as jasmonate ZIM-domain (JAZ) proteins. JAZs are key transcriptional repressors of the jasmonate (JA) signaling pathway and major components of the JA receptor complex [18], [19], [20]. JA plays an important role in regulating plant responses to biotic and abiotic stresses. Some *P. syringae* strains produce the JA-mimicking phytotoxin coronatine, which efficiently activates JA signaling to facilitate bacterial entry into plant apoplastic space and suppress defense [21], [22], [23]. Therefore, HopZ1a may also target the JAZ proteins to promote bacterial infection. Consistent to this hypothesis, HopZ1a was previously reported to induce the expression of the JA/ethylene marker gene *AtPDF1.2* in *Arabidopsis*, indicating that it could activate JA/ethylene signaling [24].

Here, we report that HopZ1a directly interacts with JAZ proteins of soybean and *Arabidopsis*. We show that HopZ1a induces the degradation of AtJAZ1, and promotes JA-responsive gene expression during *P. syringae* infection. Furthermore, HopZ1a functionally complements the growth deficiency of a *P. syringae* pv. *tomato* mutant that does not produce coronatine. All these activities depend on the intact catalytic core of HopZ1a, which acetylates JAZ proteins in vitro. Taken together, our results suggest that HopZ1a facilitates bacterial infection by manipulating the JA signaling pathway in *Arabidopsis*.

## Results

### HopZ1a physically interacts with GmJAZ1

Using yeast two-hybrid screens, we identified the HopZ1a-interacting proteins (ZINPs) from a soybean cDNA library [17]. Among them, ZINP3 (Gm7g04630) was interesting because it shows significant homology to the Jasmonate ZIM-domain (JAZ) proteins. We designated ZINP3 as GmJAZ1 because it is most similar (51% similarity in full-length amino acid sequences and 62% similarity in the ZIM and Jas domains) to AtJAZ1 in *Arabidopsis*. GmJAZ1 was then further pursued as a direct target of HopZ1a.

**Figure 1:**
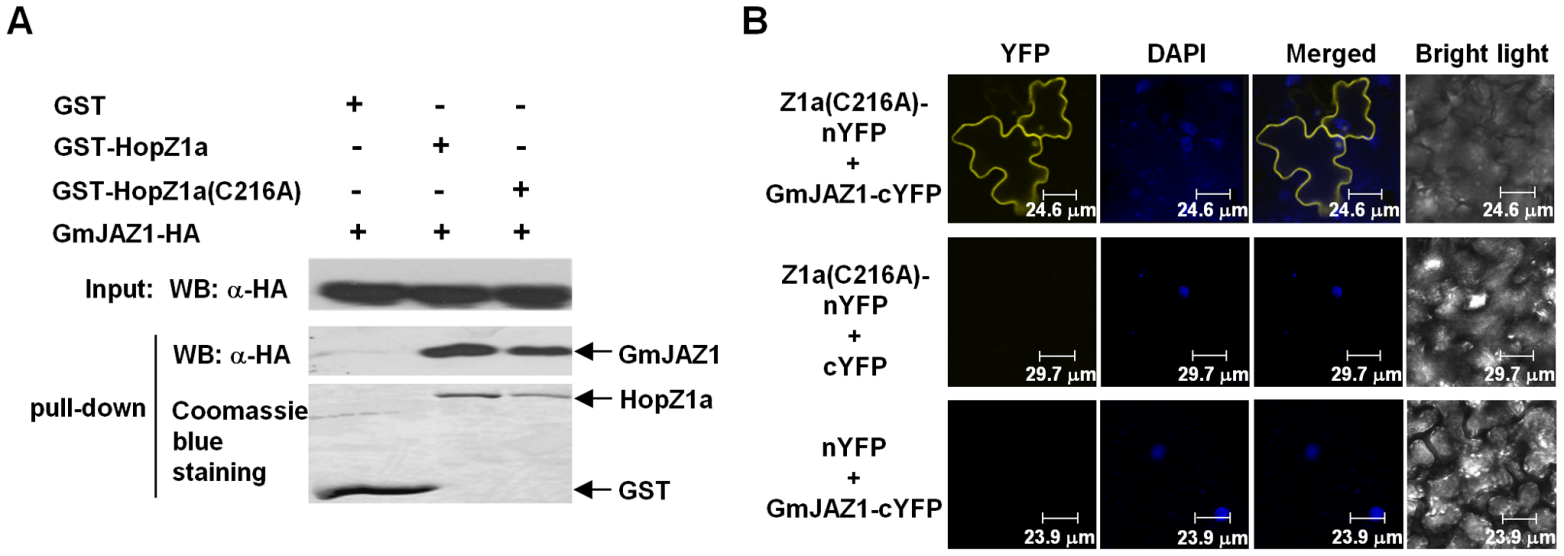
HopZ1a interacts with the soybean protein GmJAZ1. (**A**) HopZ1a and GmJAZ1 interact in vitro. GmJAZ1-HA and GST-HopZ1a proteins were expressed in *E. coli*. Precipitation of GmJAZ1 with HopZ1a was determined by western blots before (Input) and after affinity purification (Pull-down) using anti-HA antibody. The protein abundances of GST, GST-HopZ1a and GST-HopZ1a(C216A) on the affinity resins after washes were detected by Coomassie blue staining. (**B**) Bimolecular fluorescence complementation analysis showing HopZ1a-GmJAZ1 interactions in plant cells. HopZ1a(C216A)-nYFP and GmJAZ1-cYFP were co-expressed in *N. benthamiana* using *Agrobacterium*-mediated transient expression. Leaves co-infiltrated with *Agrobacterium* carrying GmJAZ1-cYFP+nYFP or cYFP+HopZ1a(C216A)-nYFP were used as negative controls. Fluorescence was detected by confocal microscopy from the infiltrated tissues at 48 hpi. DAPI was used to stain the nuclei. These experiments were repeated three times with similar results.

We first confirmed the physical interaction between HopZ1a and GmJAZ1 by in vitro pull-down using recombinant GST-HopZ1a and GmJAZ1-HA proteins over-expressed in *E. coli*. GST-HopZ1a or GST (empty vector) was purified from whole cell lysate using glutathione resins and then incubated with an equal amount of whole cell lysate of *E. coli* expressing GmJAZ1-HA. GST-HopZ1a-bound resins, but not GST-bound resins, provided enrichment of GmJAZ1-HA (Fig. 1A), suggesting that HopZ1a interacted with GmJAZ1 in vitro. The catalytic mutant HopZ1a(C216A) also interacted with GmJAZ1, similar to wild-type HopZ1a (Fig. 1A).

We next examined the sub-cellular localization of GmJAZ1 to determine whether it co-localizes with HopZ1a in plant cells. GmJAZ1-YFP was expressed in *Nicotiana benthamiana* using *Agrobacterium*-mediated transient expression. Yellow fluorescence was examined in the pavement cells of the infiltrated leaves at 48 hours post inoculation (hpi) using confocal microscopy. Fluorescence was detected both on the plasma membrane and in the nucleus (Fig. S1). Previous studies reported that HopZ1a mainly locates on the plasma membrane with a sub-pool of HopZ1a in the nucleus [17]. These results suggest that GmJAZ1 and HopZ1a could co-localize in plant cells. To further confirm that HopZ1a indeed enters the nucleus, we performed nuclear fractionation of *N. benthamiana* cells expressing HopZ1a(C216A). The catalytic mutant HopZ1a(C216A) was used in this experiment, because the expression of the functional HopZ1a triggers cell death in *N. benthamiana* [9], [14]. Consistent with the previous confocal microscopy data [17], we detected the presence of HopZ1a(C216A) from both cytosolic and nuclear fractions (Fig. S2). These data confirmed that HopZ1a and GmJAZ1 co-localize in *N. benthamiana* cells.

We further used the bimolecular fluorescence complementation (BiFC) assay to determine the interaction between HopZ1a and GmJAZ1 in planta. HopZ1a(C216A) and GmJAZ1 were fused to the nonfluorescent N-terminal domain of YFP (1–155 aa, nYFP) and the C-terminal domain of YFP (156–239 aa, cYFP), respectively, at their C-termini. When the fusion genes were co-expressed in *N. benthamiana*, fluorescence was detected on the plasma membrane and in the nucleus (Fig. 1B), consistent with the subcellular localization of GmJAZ1 and HopZ1a. Taken together, these experiments demonstrate the interaction of HopZ1a and GmJAZ1 in vitro and in planta.

### Interaction with HopZ1a leads to the degradation of GmJAZ1

**Figure 2:**
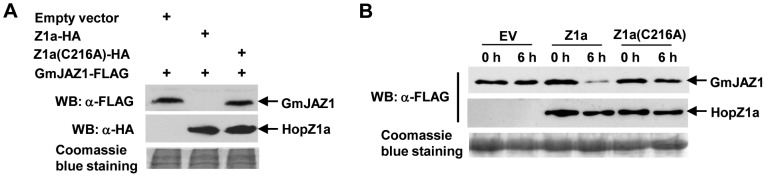
HopZ1a promotes GmJAZ1 degradation in planta. (**A**) HopZ1a induces the degradation of GmJAZ1 when the proteins were co-expressed in *N. benthamiana*. GmJAZ1-FLAG and HopZ1a-HA were transiently expressed in *N. benthamiana* and the abundance of GmJAZ1 was detected by western blots at 20 hpi. The same protein gel was stained with Coomassie blue to show equal loading. (**B**) HopZ1a induces GmJAZ1 degradation using a semi-in vitro assay. GmJAZ1-FLAG and 3×FLAG-HopZ1a were transiently expressed in *N. benthamiana* individually. Total proteins were extracted from the infiltrated leaves 20 hours post *Agro*-infiltration, mixed in equal volume, and incubated at 4°C for six hours. The abundance of GmJAZ1-FLAG was then analyzed by western blots. These experiments were repeated three times with similar results.

We have previously observed HopZ1-mediated degradation of another HopZ1-interacting protein GmHID1 when GmHID1 and HopZ1 were transiently co-expressed in *N. benthamiana* [17]. Therefore, we examined whether HopZ1a can also induce the degradation of GmJAZ1. GmJAZ1-FLAG and HopZ1a-HA were co-expressed in *N. benthamiana*, and the abundance of GmJAZ1 was determined at 20 hpi before the onset of visible cell death symptoms, which usually starts at 30 hpi. We chose 20 hpi because the expression level of GmJAZ1 was too low for protein analysis at earlier time points. A significant reduction of GmJAZ1 protein level was observed in *N. benthamiana* leaves co-expressing wild-type HopZ1a-HA, compared to leaves expressing the HopZ1a catalytic mutant or infiltrated with *Agrobacterium* carrying the empty vector (Fig. 2A). These results suggest that HopZ1a induces the degradation of GmJAZ1 in plant cells and the degradation requires the enzymatic activity of HopZ1a.

Incubation of GmJAZ1 and HopZ1a proteins purified from *E. coli* did not lead to observable changes in the abundance of GmJAZ1 (Fig. 1A). We suspected that a plant factor(s) might be required for this process and therefore performed a semi-in vitro degradation assay by incubating proteins extracted from *N. benthamiana* tissues expressing GmJAZ1 or HopZ1a individually. Total proteins extracted from leaves expressing GmJAZ1 or HopZ1a were mixed and incubated at 4°C for six hours before the abundance of GmJAZ1 was examined using western blots. Again, a significant decrease in GmJAZ1 protein level was observed in the presence of wild-type HopZ1a, but not the catalytic mutant HopZ1a(C216A) (Fig. 2B). These data suggest that HopZ1a induces GmJAZ1 degradation in plant cells.

To exclude the possibility that the reduced GmJAZ1 protein levels might have been resulted from cell death triggered by wild-type HopZ1a in *N. benthamiana*, we performed two control experiments. Firstly, we co-expressed the green fluorescence protein (GFP) with HopZ1a-HA or HopZ1a(C216A)-HA in *N. benthamiana*. The GFP protein levels remained unchanged in the presence of either wild-type or the catalytic mutant of HopZ1a (Fig. S3A). Secondly, we performed the semi-in vitro degradation assay of GmJAZ1 using AvrRpt2, which also elicits cell death in *N. benthamiana* [25]. Incubation with plant protein extracts expressing AvrRpt2 did not change the abundance of GmJAZ1 (Fig. S3B). This suggests that the reduced abundance of GmJAZ1 was not a result of HopZ1a-induced cell death in *N. benthamiana*.

### HopZ1a physically interacts with *Arabidopsis* JAZs

**Figure 3:**
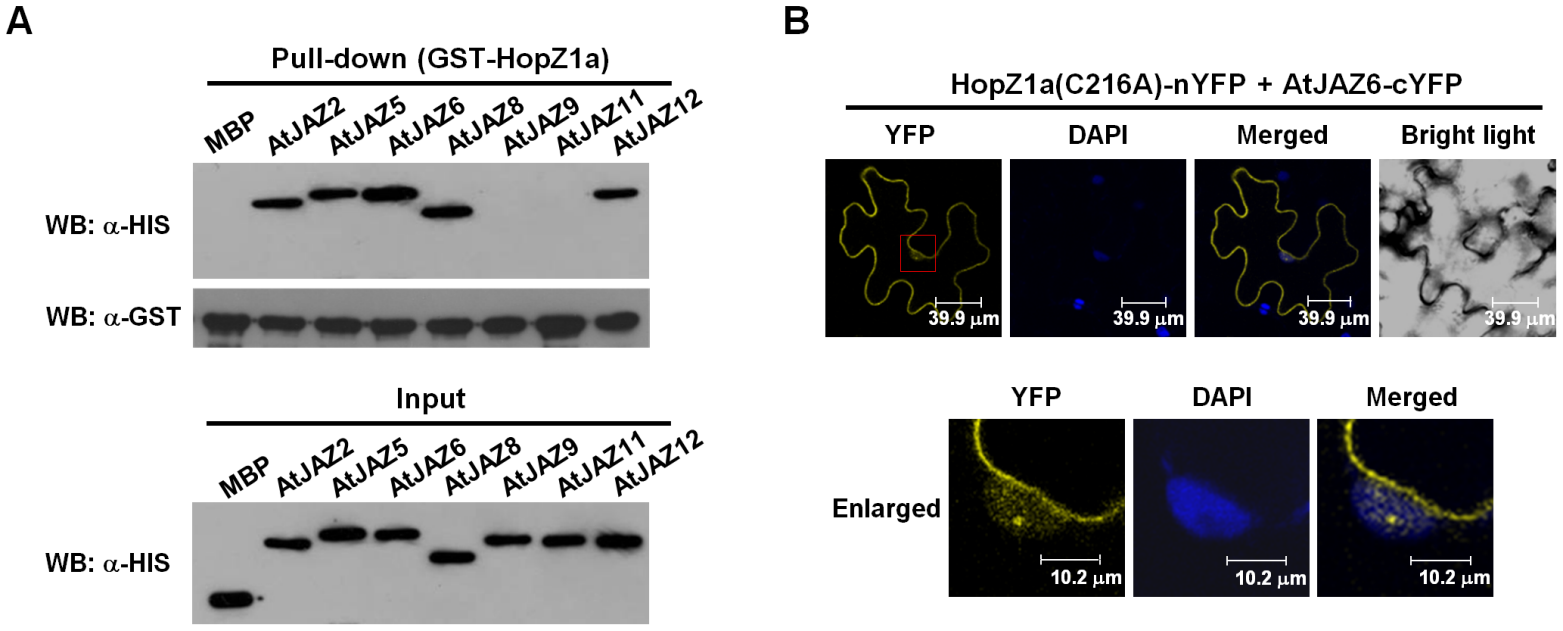
HopZ1a interacts with AtJAZs. (**A**) HopZ1a interacts with AtJAZs in vitro. Precipitation of MBP-AtJAZ-HIS with GST-HopZ1a was detected by western blots before (Input) and after affinity purification (Pull-down) using anti-HIS antibody. (**B**) Bimolecular fluorescence complementation analysis showing the interaction between HopZ1a and AtJAZ6 in planta. HopZ1a(C216A)-nYFP and AtJAZ6-cYFP were co-expressed in *N. benthamiana*. Fluorescence in the infiltrated leaves was monitored by confocal microscopy at 48 hours post *Agro*-infiltration. DAPI was used to stain the nuclei. These experiments were repeated three times with similar results.

Because GmJAZ1 is an ortholog of *Arabidopsis* JAZ proteins (AtJAZs), we examined whether HopZ1a also targets AtJAZs. *Arabidopsis* produces twelve JAZ orthologs (Fig. S4). Among them, seven were tested for their interactions with HopZ1a using in vitro pull-down. Our data showed that AtJAZ2, AtJAZ5, AtJAZ6, AtJAZ8 and AtJAZ12 interacted with HopZ1a in vitro (Fig. 3A). Although AtJAZ1 shares the highest sequence similarity with GmJAZ1 (Fig. S4), the interaction of AtJAZ1 with HopZ1a could not be determined because we were unable to express AtJAZ1 in *E. coli* at a level suitable for the pull-down assay.

We next confirmed the interaction between HopZ1a and AtJAZ6 in planta using BiFC. Similar to HopZ1a-GmJAZ1 interaction, yellow fluorescence was observed from plasma membrane and nucleus in cells co-expressing HopZ1a(C216A)-nYFP and AtJAZ6-cYFP (Fig. 3B). AtJAZ6 by itself was mainly located in the nucleus, but could also be detected in cytosol (Fig. S2). These data suggest that HopZ1a and AtJAZ6 co-localize and interact in plant cells.

### HopZ1a acetylates JAZs in vitro

**Figure 4:**
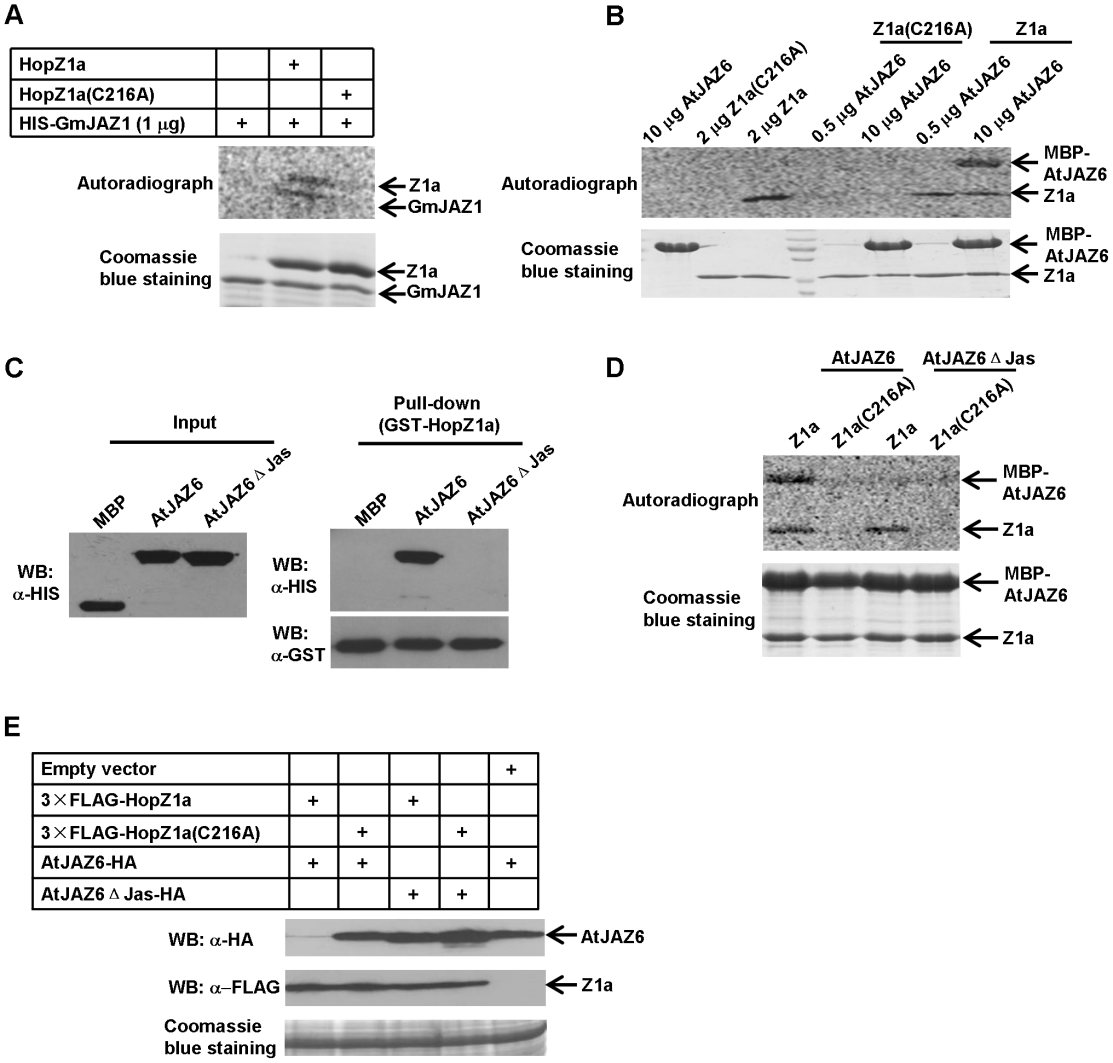
JAZs are acetylation substrates of HopZ1a. (**A**) HopZ1a acetylates GmJAZ1 in vitro. Tag-free HopZ1a and HopZ1a(C216A), and HIS-GmJAZ1 were purified from *E. coli* and subjected to in vitro acetylation assays. The acetylated proteins were detected by autoradiography after exposure at −80°C for five days. (**B**) HopZ1a acetylates MBP-AtJAZ6-HIS in vitro. (**C**) The mutant AtJAZ6ΔJas no longer interacts with HopZ1a. Purified HopZ1a was incubated with MBP-AtJAZ6-HIS or MBP-AtJAZ6ΔJas-HIS in the in vitro pull-down assay. (**D**) HopZ1a no longer acetylates AtJAZ6ΔJas. (**E**) HopZ1a does not trigger the degradation of AtJAZ6ΔJas. AtJAZ6ΔJas-YFP-HA and 3×FLAG-HopZ1a were co-expressed in *N. benthamiana*. The abundance of AtJAZ6ΔJas was detected by western blots. All the in vitro acetylation experiments were repeated at least three times with similar results. The in vitro pull-down and degradation experiments were repeated twice with similar results.

Several effectors from the YopJ family, including HopZ1a, have been shown to possess acetyltransferase activities. To determine whether JAZs are substrates of HopZ1a, we performed in vitro enzymatic assay using C14-labeled acetyl-CoA. Recombinant HIS-SUMO-HopZ1a or HIS-SUMO-HopZ1a(C216A) proteins were expressed in *E. coli* and purified using nickel column. The HIS-SUMO tag was then removed by ubiquitin like protease 1 (ULP1). Tag-free HopZ1a or HopZ1a(C216A) proteins were incubated with purified HIS-GmJAZ1or MBP-AtJAZ6-HIS proteins in the presence of the cofactor inositol hexakisphosphate (IP6), and the acetylation of HopZ1a, GmJAZ1 and AtJAZ6 was detected by autoradiography as previously described [26]. Our experiments showed that both GmJAZ1 (Fig. 4A) and AtJAZ6 (Fig. 4B) were acetylated by wild-type HopZ1a, which also exhibited autoacetylation. The acetylation of GmJAZ1 appeared to be weaker in the autoradiograph compared to that of AtJAZ6. This is due to the low expression level of GmJAZ1 in *E. coli*, which only allowed us to use a much lower amount (1 µg), compared to AtJAZ6 (10 µg) in the reactions. Nonetheless, we consistently detected the acetylated form of GmJAZ1 when it was incubated with HopZ1a, but not HopZ1a(C216A), suggesting that GmJAZ1 and AtJAZ6 are both substrates of HopZ1a.

We sometimes could observe a background level of acetylation in tagged AtJAZ6 (MBP-AtJAZ6-HIS) when it was incubated with HopZ1a(C216A). Although this background acetylation was very weak compared to the acetylation of MBP-AtJAZ6-HIS, we decided to use the tag-free AtJAZ6 proteins to further confirm its acetylation by HopZ1a. Again, we observed strong acetylation of AtJAZ6 by HopZ1a, but not by HopZ1a(C216A) using only 5 µg of AtJAZ6 in the reaction (Fig. S5). These results demonstrate that GmJAZ1 and AtJAZ6 are acetylation substrates of HopZ1a.

### The Jas domain of AtJAZ6 is required for HopZ1a interaction

JAZ proteins share three conserved domains: the C-terminal Jas motif [27], the ZIM domain in the central region [28], and a weakly conserved N-terminal region [19]. Because the conserved Jas domain is essential for the instability of JAZs in response to JA and the JA-mimicking phytotoxin coronatine [18], [19], we examined the impact of the Jas domain in the interaction between JAZs and HopZ1a. We constructed the mutant AtJAZ6ΔJas by deleting ten highly conserved amino acids (from seine^191^ to lysine^200^) within the Jas domain. In vitro pull-down assay showed that AtJAZ6ΔJas did not bind HopZ1a (Fig. 4C). Furthermore, AtJAZ6ΔJas was not acetylated by HopZ1a in vitro (Fig. 4D) or degraded by HopZ1a when these two proteins were co-expressed in *N. benthamiana* (Fig. 4E). These results demonstrate that HopZ1a-induced JAZ degradation requires direct interaction of HopZ1a with AtJAZ6, which is mediated by the Jas domain.

### HopZ1a triggers the degradation of AtJAZ1 during bacterial infection

**Figure 5:**
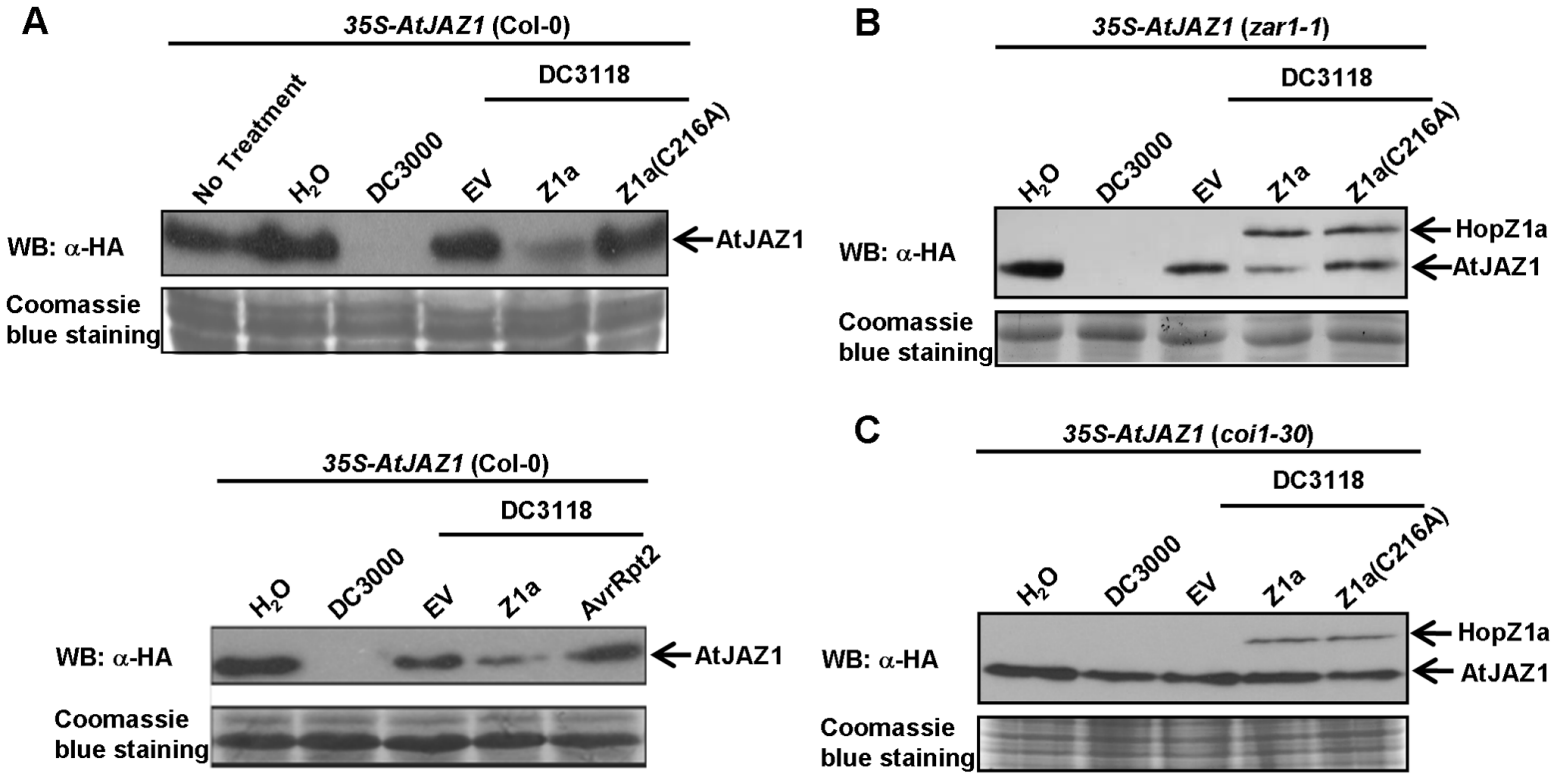
HopZ1a triggers the degradation of AtJAZ1 during bacterial infection. (**A**) HopZ1a, but not AvrRpt2, promotes the degradation of AtJAZ1 in the *Arabidopsis* ecotype Col-0 (wild-type) during bacterial infection. Six-week *35S*-*HA-AtJAZ1Arabidopsis* transgenic plants were infiltrated with *Pto*DC3000, *Pto*DC3118 carrying the empty pUCP18 vector (EV), or *Pto*DC3118 expressing HopZ1a, HopZ1a(C216A) or AvrRpt2. (**B**) HopZ1a promotes the degradation of AtJAZ1 in *zar1-1 Arabidopsis* plants. Six week-old *35S*-*HA-AtJAZ1 zar1-1 Arabidopsis* plants were inoculated with *Pto*DC3000, *Pto*DC3118 carrying the empty pUCP18 vector (EV), or *Pto*DC3118 expressing HopZ1a or HopZ1a(C216A). (**C**) HopZ1a-mediated degradation of AtJAZ1 is dependent on COI1. *35S*-*HA-AtJAZ1*, *coi1-30 Arabidopsis* plants were inoculated with *Pto*DC3000, *Pto*DC3118 carrying the empty pUCP18 vector (EV), or *Pto*DC3118 expressing HopZ1a or HopZ1a(C216A). Bacterial infection assays were conducted using inoculums at OD_600_ = 0.2 (approximately 2×10^8^ cfu/mL). The abundance of AtJAZ1 was determined by western blots using anti-HA antibody at 6 hpi. The protein gels were stained with Coomassie blue as loading controls. These experiments were repeated three times with similar results.

Although we observed the degradation of GmJAZ1 and AtJAZ6 when they were co-expressed with HopZ1a in *N. benthamiana*, it is important to examine whether HopZ1a can promote JAZ degradation during bacterial infection. For this purpose, we inoculated transgenic *Arabidopsis* plants expressing *35S*-*HA-AtJAZ1* with *P. syringae* producing HopZ1a or HopZ1a(C216A). The *Arabidopsis* pathogen *Pseudomonas syringae* pv. *tomato* strain DC3000 (*Pto*DC3000) is well-known to induce AtJAZ degradation through the production of coronatine, which acts as a JA mimic [22]. The mutant *Pto*DC3118 is deficient in coronatine production and therefore no longer degrades JAZs [29]. Importantly, *Pto*DC3118 expressing HopZ1a from its native promoter also significantly reduced the abundance of AtJAZ1 at 6 hpi (Fig. 5A). The level of AtJAZ1 remained unchanged in tissues infiltrated with *Pto*DC3118 carrying the empty vector or expressing the catalytic mutant HopZ1a(C216A). These data strongly suggest that HopZ1a can induce AtJAZ1 degradation during bacterial infection.

Because HopZ1a elicits HR in *Arabidopsis* ecotype Col-0, we performed two experiments to exclude the possibility that HopZ1a-triggered AtJAZ1 degradation was a result of plant cell death. First, we examined whether another effector AvrRpt2 could induce AtJAZ1 degradation. Although AvrRpt2 also triggers HR in *Arabidopsis* Col-0, the abundance of AtJAZ1 was unchanged when the *HA-AtJAZ1*-expressing plants were inoculated with *Pto*DC3118 expressing AvrRpt2 (Fig. 5A). Next, we generated the transgenic *Arabidopsis* line expressing *35S*-*HA-AtJAZ1* in the *zar1-1* mutant background, which is abrogated in HopZ1a-triggered HR [30]. Again, the AtJAZ1 protein level was significantly reduced by HopZ1a (Fig. 5B), confirming that HopZ1a delivered by *P. syringae* leads to AtJAZ1 degradation in a cell death independent manner.

### HopZ1a-mediated JAZ degradation is dependent on coronatine-insensitive 1 (COI1)

A major regulatory mechanism of JAZs in the presence of JA or coronatine is through COI-dependent ubiquitin-proteasome degradation. COI1 is an F-box protein that determines the substrate specificity of a Skp/Cullin/F-box (SCF) E3 ubiquitin ligase-SCF^COI1^ [31]. Together with JAZ, COI1 is also a critical component of the JA co-receptor complex [19], [20], [22]. We examined whether COI1 is required for HopZ1a-induced JAZ degradation using the transgenic *Arabidopsis* line expressing *35S*-*HA-AtJAZ1* in the *coi1-30* (SALK_035548) mutant background. As expected, *Pto*DC3000, which induces JAZ degradation through coronatine production, was unable to reduce the abundance of AtJAZ1 in the absent of COI1. Interestingly, *Pto*DC3118 expressing HopZ1a also no longer induced the degradation of AtJAZ1 in the *coi1* mutant plants (Fig. 5C). These data suggest that, similar to coronatine- and JA-mediated AtJAZ degradation, COI1 is required for the degradation of AtJAZ1 by HopZ1a.

### HopZ1a activates JA signaling

**Figure 6:**
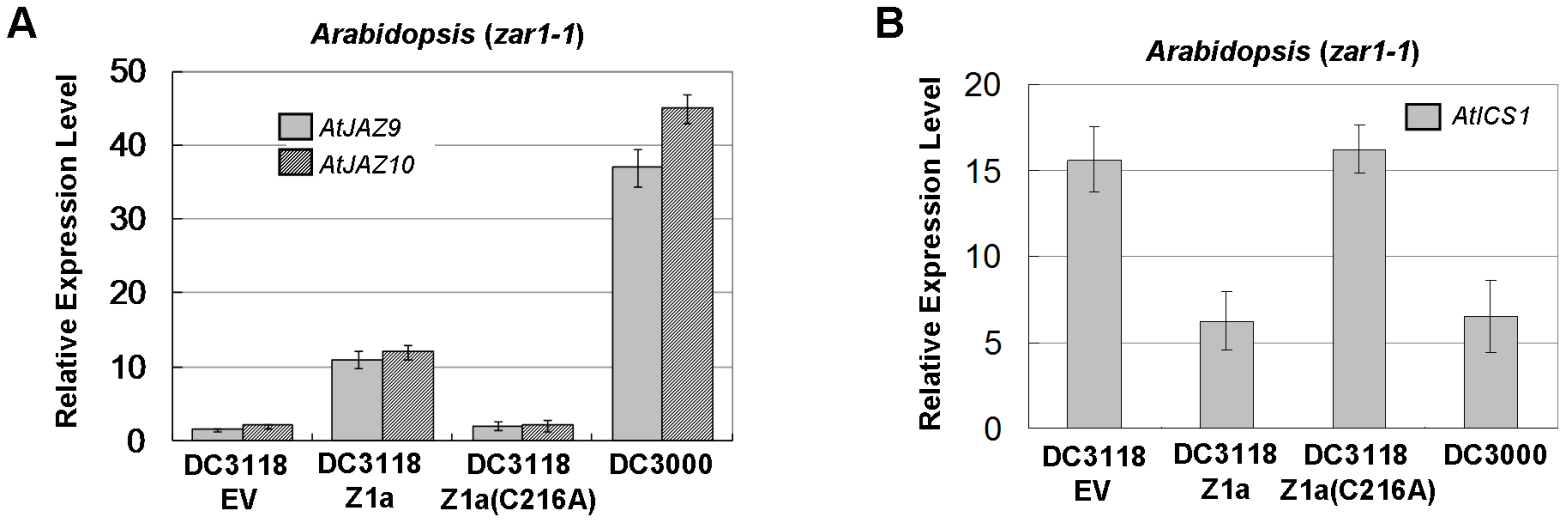
HopZ1a activates JA signaling during bacterial infection. *Arabidopsis zar1-1* mutant plants were inoculated with *Pto*DC3000 or *Pto*DC3118 carrying the empty pUCP18 vector (EV), HopZ1a or HopZ1a(C216A). The transcript levels of the JA-responsive genes *AtJAZ9* and *AtJAZ10*, as well as the SA biosynthetic gene *AtICS1* were determined by quantitative RT-PCR. (**A**) HopZ1a induces the expression of JA-responsive genes in *Arabidopsis*. The abundances of *AtJAZ9* and *AtJAZ10* transcripts were examined at 6 hpi using *AtActin* as the internal standard. Relative expression levels were determined by comparing the normalized *AtJAZ9* or *AtJAZ10* transcripts between infected and mock-treated (leaves infiltrated with 10 mM MgSO_4_) samples. (**B**) HopZ1a reduces the expression of *AtICS1* in *Arabidopsis*. *AtICS1* transcript level was analyzed at 9 hpi using *AtUBQ5* as the internal standard. Values are means ± standard deviations (as error bars) (n = 5). All experiments were repeated at least five times with similar results. The expression of HopZ1a in *P. syringae* was confirmed by western blots (Fig. S8).

In *Arabidopsis*, JAZ proteins are repressors of JA transcription factors (e.g. AtMYC2) that are involved in the expression of JA-responsive genes [32], [33], [34]. Since HopZ1a induces the degradation of AtJAZ1, we examined whether it could induce the expression of JA-responsive genes during bacterial infection. Real-time RT-PCR was carried out to determine the transcript levels of JA-responsive genes in *Arabidopsis*. Five-week old *zar1-1* plants were inoculated with *Pto*DC3118 expressing HopZ1a or HopZ1a(C216A) at OD_600_ = 0.2 (approximately 2×10^8^ cfu/mL). The transcript levels of two early JA-responsive genes, *AtJAZ9* and *AtJAZ10* [34], were analyzed at 6 hpi. Both genes were induced approximately ten fold in plants infected by *Pto*DC3118(HopZ1a), whereas their expression was not changed in tissues infected by *Pto*DC3118 expressing HopZ1a(C216A) (Fig. 6A). The level of gene induction by HopZ1a was lower than that by coronatine, as shown by the approximately 40-fold induction of *AtJAZ9* and *AtJAZ10* in plants infected with *Pto*DC3000. This is consistent with the partial vs. complete degradation of AtJAZ1 by HopZ1a or coronatine during bacterial infection. Nonetheless, these experiments suggest that bacterium-delivered HopZ1a can activate JA signaling.

Recent findings showed that coronatine can suppress salicylic acid (SA) accumulation, probably as a consequence of the activation of JA signaling [35]. Because SA-associated defense confers resistance against biotrophic and hemibiotrophic pathogens, reduced SA accumulation would lead to defense suppression. In particular, coronatine is able to repress the expression of the SA synthetic enzyme isochorismate synthase gene 1 (*AtICS1*) in *Arabidopsis*. We then examined the impact of HopZ1a on the expression of *AtICS1*. *Arabidopsis zar1-1* plants were inoculated with *Pto*DC3000 or *Pto*DC3118 carrying empty vector, HopZ1a, or HopZ1a(C216A) at OD_600_ = 0.2 (approximately 2×10^8^ cfu/mL). Consistent with the prior findings, the transcript abundance of *AtICS1* was reduced in plants infected with *Pto*DC3000 when compared to *Pto*DC3118 carrying the empty vector or HopZ1a(C216A) (Fig. 6B). The expression of *AtICS1* was also reduced in plants inoculated with *Pto*DC3118 expressing HopZ1a, to a similar level as that in leaves inoculated with *Pto*DC3000. These data confirmed that, like coronatine, HopZ1a activates JA signaling and represses SA accumulation during bacterial infection.

### HopZ1a facilitates bacterial multiplication in *Arabidopsis* in a COI1-dependent manner

**Figure 7:**
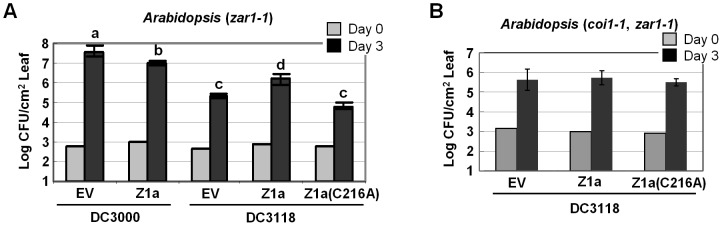
HopZ1a partially complements the virulence function of coronatine in bacteria growth. (**A**) HopZ1a promotes the multiplication of *Pto*DC3118 in *Arabidopsis*. *Arabidopsis zar1-1* plants were dip-inoculated with *Pto*DC3000 carrying pUCP20tk (EV), pUCP20tk::*hopZ1a-HA*, or *Pto*DC3118 carrying pUCP18 (EV), pUCP18::*hopZ1a-HA* or pUCP18::*hopZ1a(C216A)-HA* at OD_600_ = 0.2 (approximately 2×10^8^ cfu/mL). Bacterial populations were determined at 0 and 3 days post inoculation. The average colony forming units per square centimeter (cfu/cm^2^) and standard deviations (as error bars) are presented. Different letters at the top of the bars represent data with statistically significant differences (two tailed t-test *p*<0.01). (**B**) COI1 is required for the virulence activity of HopZ1a. *coi1-1, zar1-1* double mutant plants were dip-inoculated with *Pto*DC3118 carrying pUCP18 (EV), pUCP18::*hopZ1a-HA* or pUCP18::*hopZ1a(C216A)-HA*. Bacterial multiplications were examined at 0 and 3 days post inoculation. The average colony forming units per square centimeter (cfu/cm^2^) and standard deviations (as error bars) are presented. The expression of HopZ1a or HopZ1a(C216A) in *P. syringae* was confirmed by western blots (Fig. S8). These experiments were repeated at least five times with similar results.

Coronatine facilitates the infection of *Pto*DC3000 by activating JA signaling in *Arabidopsis* [21]. The coronatine-deficient mutant *Pto*DC3118 exhibits a significant reduction in bacterial population especially when the plants are infected by dipping inoculation [36]. Since HopZ1a also activates JA signaling, we examined whether HopZ1a could complement the growth deficiency of *Pto*DC3118. The *Arabidopsis zar1-1* mutant plants were dipping-inoculated by *Pto*DC3000 or *Pto*DC3118 carrying the empty vector, HopZ1a, or HopZ1a(C216A). Three days post infection (dpi), the bacterial populations of *Pto*DC3118 carrying the empty vector or expressing HopZ1a(C216A) were approximately 200 fold lower than that of *Pto*DC3000 (Fig. 7A). Importantly, *Pto*DC3118 expressing wild-type HopZ1a multiplied to a significantly higher level (about 10 fold) than *Pto*DC3118 or *Pto*DC3118 expressing HopZ1a(C216A) (Fig. 7A). Although the population of *Pto*DC3118(HopZ1a) is lower than that of *Pto*DC3000, this partial complementation of the growth deficiency of *Pto*DC3118 is consistent with the partial degradation of AtJAZ1 (Fig. 5A) and the lower levels of JA-responsive gene induction (Fig. 6A) by *Pto*DC3118(HopZ1a) compared to *Pto*DC3000.

To further confirm that the function of HopZ1a is specifically related to its ability to activate the JA pathway, we introduced HopZ1a into *Pto*DC3000 and performed the same bacterial growth assay. HopZ1a was previously shown to enhance the infection of *Pto*DC3000 [30]. However, despite numerous trials, we did not observe any enhancement of HopZ1a on in planta multiplication of *Pto*DC3000. In fact, we consistently detected a decrease in the population of *Pto*DC3000(HopZ1a) compared to *Pto*DC3000 carrying the empty vector (Fig. 7A). Thus, HopZ1a can partially substitute for coronatine to promote bacterial infection.

Because COI1 is required for HopZ1a-induced degradation of AtJAZ1, we then examined whether COI1 is also required for the virulence activity of HopZ1a in *Arabidopsis*. For this purpose, we generated *coi1-1, zar1-1* double mutant *Arabidopsis* plants, which were inoculated by *Pto*DC3118 carrying the empty vector, HopZ1a or HopZ1a(C216A). The bacterial populations of these three strains remained the same (Fig. 7B), confirming that the virulence activity of HopZ1a requires COI1. To further confirm that HopZ1a was unable to activate JA signaling in the mutant plants, we also determined the transcript levels of the JA-responsive genes *AtJAZ9* and *AtJAZ10*, as well as the SA-biosynthetic gene *AtICS1* after bacterial inoculation. Similar to *Pto*DC3000, *Pto*DC3118 expressing HopZ1a was also unable to induce the expression of *AtJAZ9* and *AtJAZ10* or suppress the expression of *AtICS1* (Fig. S6). These data suggest that both the phytotoxin coronatine and the effector HopZ1a activate JA signaling in a COI1-dependant manner.

HopZ1a contains a potential N-terminal myristoylation site (Gly2) which may direct the association of HopZ1a with plasma membrane in plant cells [14], [15]. We therefore examined whether the Gly2 residue was required for the virulence function of HopZ1a. The bacterial population of *Pto*DC3118 carrying HopZ1a(G2A) was similar to that of *Pto*D3118 carrying wild-type HopZ1a in *Arabidopsis zar1-1* plants (Fig. S7). This result demonstrates that HopZ1a(G2A) retained the virulence activity to promote *Pto*D3118 multiplication in *Arabidopsis*, indicating that the potential myristoylation site is not required for the virulence function of HopZ1a.

Taken together, our experiments showed that HopZ1a enhanced *P. syringae* infection in *Arabidopsis* in a manner similar to coronatine, which is a potent activator of JA signaling during bacterial infection.

## Discussion

T3SEs manipulate a variety of cellular processes in eukaryotic hosts for the benefit of pathogen infection. Emerging data suggest that bacterial pathogens have evolved various effectors to manipulate the signaling of JA and SA, which are important plant hormones that regulate defense response [37]. SA-dependent defense plays a major role in plant immunity against biotrophic and hemibiotrophic pathogens, such as *Hyaloperonospora arabidopsidis* and *P. syringae* [35], [37]. The *P. syringae* effector HopI1 directly targets Hsp70 in choloroplasts to suppress SA accumulation and thereby facilitate bacterial infection [38]. In addition, the *Xanthomonas campestris* effector XopD was also shown to repress SA signaling during bacterial infection of tomato [39], [40], [41]. Here, we report that the *P. syringae* effector HopZ1a represses SA accumulation, probably as an indirect effect of the activation of JA signaling.

In this study, we report that HopZ1 directly targets JAZs, the key negative regulators of JA signaling [18], [19]. Because JA signaling pathway is antagonistic to SA-dependent defense, activating JA signaling would be an attractive bacterial strategy to suppress host defense and facilitate pathogenesis of these pathogens. Indeed, recent studies have shown a remarkable example in which the *P. syringae* phytotoxin coronatine structurally mimics the active form of JA and targets the JAZ repressors for degradation to efficiently activate JA signaling [20], [22]. However, it has been rather puzzling that only a few *P. syringae* strains produce coronatine [42]. A previous study showed that a T3SE, AvrB, was also able to promote JA signaling, apparently through an indirect mechanism via the activation of MPK4 [43], [44]. We show here that the effector HopZ1a directly interacts with JAZs and at least some JAZs can be used by HopZ1a as substrates for acetylation. Importantly, HopZ1 mediates the degradation of AtJAZ1 in *Arabidopsis* and promotes bacterial infection in a COI1-dependent manner. This new finding raises the exciting possibility that JAZ repressors (hence the JA receptor complex) may be a common hub of host targets for diverse bacterial virulence factors. Consistent with this notion, oomycete pathogens were also found to produce effectors that interact with AtJAZ3 [45]. These pieces of evidence suggest that the JA receptor complex might be the Achilles' heel in plant defense system during the arms race with microbial pathogens.

HopZ1a enhances the in planta multiplication of *Pto*DC3118, but not that of *Pto*DC3000, in *Arabidopsis*. A weak growth enhancement of *Pto*DC3000 by HopZ1a was reported previously [30]. We were unable to replicate the published data, probably due to differences in experimental conditions. Our experiments, including the JA-responsive gene expression, JAZ protein degradation and bacterial in planta multiplication, consistently suggest that HopZ1a activates the JA signaling pathway in a manner similar to coronatine. However, HopZ1a only partially complements the function of coronatine. This could be because HopZ1a is not as potent as coronatine in inducing the degradation of JAZs. Coronatine has dual functions during the pre-entry and post-entry stages of bacterial infection [23], whereas the type III secretion genes are generally believed to be expressed after the bacteria have entered the apoplast [46]. Although it remains to be determined whether HopZ1a could promote stomata opening at the pre-entry stage, it is possible that HopZ1a mainly substitutes coronatine function inside the plant tissues. In addition, proteins might not be as stable as metabolites in planta, which may also explain the partial complementation of HopZ1a for the virulence deficiency of the coronatine mutant *Pto*DC3118.

As transcription regulators, JAZs are believed to function in the nucleus. However, HopZ1a was previously shown to mainly locate on the plasma membrane and this localization was mediated by a potential myristoylation site in the N-terminus [15]. Our protein-protein interaction and localization analyses showed that HopZ1a is also located in the nucleus and it interacts with JAZs both in the nucleus and on the cytosol/plasma membrane. Importantly, the mutant HopZ1a(G2A), which is abolished for its localization on the plasma membrane, was still able to promote *Pto*DC3118 infection. These data demonstrate that the membrane localization of HopZ1a is only important for host recognition [14], [15], but not required for virulence activity. This is consistent with the primary localization of JAZs in the nucleus as transcription repressors.

YopJ-like T3SEs produced by plant pathogens appear to have various enzymatic activities. AvrXv4 of *Xanthomonas campestris* was reported to be a SUMO protease [47]. AvrBsT, also from *Xanthomonas*, exhibited a weak cysteine protease activity in vitro [48]. PopP2 in *R. solanacearum* has autoacetylation and trans-acetylation activities in vitro, but it does not seem to acetylate its host target proteins [13]. Recently, HopZ1a was demonstrated to acetylate tubulin [16]. Our experiments showed that GmJAZ1 and AtJAZ6 are also substrates of HopZ1a. Importantly, we found that HopZ1a induces the degradation of AtJAZ1 during bacterial infection. In the presence of the active form of JA or coronatine, the F-box protein COI1 interacts with JAZs via the C-terminus Jas motif and recruits JAZs to the *26S* proteasomes for degradation [18], [19], [22]. The fact that HopZ1a-mediated AtJAZ1 degradation is dependent on COI1 suggests that this degradation could also be dependent on the *26S* proteasomes as a consequence of JAZ modification by HopZ1a. Post-translational modifications, including acetylation, have been shown to induce or repress proteasomal degradation. For example, in mammalian cells, the acetyltransferase ARD1 acetylates Hypoxia-inducible factor 1α (HIF-1α), which promotes its ubiquitination and proteasomal degradation [49]. Further investigations are needed to determine how HopZ1a-mediated acetylation of JAZs could facilitate COI1-dependent degradation of JAZ repressors.

## Materials and Methods

### Bacterial strains and plasmids

*Pseudomonas syringae*, *Agrobacterium tumefaciens* and *Escherichia coli* strains were grown as described [50]. Bacteria strains and plasmids used in this study are summarized in Table S1.

### Fluorescence microscopy

To construct plasmids for bimolecular fluorescence complementation (BiFC) assay, full-length cDNA of *GmJAZ1* or *AtJAZ6* and *hopZ1a(C216A)* were cloned into the vectors pSPYCE and pSPYNE [51], respectively. To examine the subcellular localization of GmJAZ1, full-length cDNA was cloned into the vector pEG101[52]. The recombinant plasmids were introduced into *Agrobacterium tumefaciens* strain C58C1(pCH32), which were then used to infiltrate 3–4 week old *Nicotiana benthamiana* plants using a protocol described previously [14]. Functional fluorophore were visualized in the infiltrated leaves using a Leica SP2 Laser Scanning Confocal Microscope (Leica Microsystems) at 48 hpi for subcellular localization and BiFC. DAPI was used to stain the nucleus in plant cells [53], [54].

### In vitro GST pull-down assays

To construct GST-fusion plasmids, the full-length *hopZ1a* gene was inserted into the vector pGEX4T-2 (GE Healthcare Life Science). *GmJAZ1-HA* gene was cloned into the vector pET14b (Navogen), which has 6×His in the N-terminus. The *AtJAZ* genes were cloned into the plasmid vector pET-Mal with maltose binding protein (MBP) in the N-terminus and 8×His in the C-terminus [55]. In vitro pull-down assays were carried out using GST pull-down protein∶protein interaction kit (Pierce) according to the manufacturer's instruction. Briefly, GST or GST-HopZ1a was expressed in *E. coli* strain BL21(DE3). Soluble proteins were incubated with 50 µL glutathione agarose beads (Invitrogen) for one hour at 4°C. The beads were washed (20 mM Tris-HCl (PH 7.5), 150 mM KCl, 0.1 mM EDTA and 0.05% Triton X-100) five times and then incubated with equal amount of bacterial lysates containing JAZ proteins at 4°C for overnight. The beads were washed five times again, and the presence of the JAZ proteins on the beads was detected by western blots using anti-HA or anti-His antibodies conjugated with horseradish peroxidase (HRP) ) (Santa Cruz Biotechnology Inc.).

### Protein analysis

*Pseudomonas syringae* expressing the *hopZ1a-HA* genes was induced in M63 minimal medium containing 1% fructose at room temperature overnight [50]. HopZ1 expression was detected by western blots using the anti-HA antibody.

For JAZ degradation assay in *N. benthamiana*, *hopZ1a-HA* or *3*×*FLAG-hopZ1a* were co-expressed with *GmJAZ1-FLAG* or *AtJAZ6-YFP-HA* using *Agrobacterium*-mediated transient expression as previously described [9], [56]. Leaf disks were collected at 20 hpi, and then grounded in 2×Laemmli buffer. The abundances of GmJAZ1 and AtJAZ6 were analyzed by western blots.

For the semi-in vitro protein degradation assay, GmJAZ1-FLAG and 3×FLAG-HopZ1a were over-expressed individually in *N. benthamiana* using *Agrobacterium*-mediated transient expression. Total proteins were extracted from the infected leaf tissues at 20 hpi using an extraction buffer containing 200 mM NaCl, 50 mM Tris (pH 7.6), 10% Glycerol, 0.1% NP-40, protease inhibitor cocktail (Roche), 10 mM DTT, 1 mM PMSF. Protein extracts were mixed in equal volume for six hours at 4°C with gentle agitation before the abundance of GmJAZ1 was examined by western blots.

For the in vivo JAZ degradation assay, six-week-old *35S*-*HA-AtJAZ1* transgenic *Arabidopsis* plants were hand infiltrated with bacterial suspensions of *Pto*DC3000 or *Pto*DC3118 carrying the empty vector (EV), expressing HopZ1a, HopZ1a(C216A) or AvrRpt2 at OD_600_ = 0.2 (approximately 2×10^8^ cfu/mL). Leaves infiltrated with water were used as a negative control. Six hours post inoculation, total proteins were extracted from four leaf discs (0.5 cm^2^) in 100 µL of 2×SDS sample buffer. The lysates were incubated at 95°C for 10 minutes followed by centrifugation at 14,000 rpm for 5 minutes. The abundance of AtJAZ1 was then analyzed by western blots. Homozygous *coi1-30* mutant plants were selected on 1/2×Murashige & Skoog (MS) medium supplemented with 50 µM JA. Seedlings that were insensitive to JA treatment, i.e. without inhibited root growth symptoms, were transplanted in soil and infected with *P. syringae* after six weeks.

### In vitro acetylation assays

HIS-GmJAZ1, HIS-SUMO-HopZ1a, HIS-SUMO-HopZ1a(C216A), HIS-SUMO-AtJAZ6, MBP-AtJAZ6-HIS, and MBP-AtJAZ6ΔJas-HIS were over-expressed in the *E. coli* strain BL21(DE3) and then purified using nickel resins. HIS-SUMO-HopZ1a and HIS-SUMO-AtJAZ6 proteins were then cleaved by ULP1 protease, producing protein mixtures with both HIS-SUMO and either tag-free HopZ1a or AtJAZ6. The protein mixtures were incubated with nickel resin again and the tag-free HopZ1a or AtJAZ6 proteins were collected from the flow through. In a standard acetylation assay, 2 µg purified HopZ1a or HopZ1a(C216A) was incubated with 10 µg MBP-AtJAZ6, 5 µg AtJAZ6 or 1 µg GmJAZ1 at 30°C for one hour in 25 µL of reaction buffer containing 50 mM HEPES (pH 8.0), 10% glycerol, 1 mM DTT, 1 mM PMSF, 10 mM sodium butyrate, 1 µL [14C]-acetyl-CoA (55 µci/µmol,) and 100 nM IP6. The reaction mixtures were then subjected to SDS-PAGE and acetylated proteins were detected by autoradiography as previously described [11], [26], [57] after exposure at −80°C for five days. After autoradiograph, the protein gels were removed from the filter paper and stained with Coomassie blue as a loading control.

### Real time RT-PCR

The transcript abundances of *AtJAZ9*, *AtJAZ10* or *AtICS1* in *Arabidopsis* leaf tissues were analyzed by real-time RT-PCR using SYBR Green Supermix (BioRad Laboratories) and an CFX96 Real-Time PCR Detection System (BioRad Laboratories). Total RNA was isolated from three independent biological replicates using Trizol, and DNA was removed using DNase I (Fermentas). Reverse transcription was performed using M-MLV Reverse Transcriptase (Promega) with 1 µg of total RNA in a 25 µL reaction. The cDNAs were then used as templates for real-time PCR using gene-specific primers, which are listed below. *AtActin* was used as internal standard when compared the expression of *AtJAZ9 and AtJAZ10* in different treatment. *AtUBQ5* was the internal control used for the normalization of *AtICS1* expression.

*AtActin*: 5′-GGTGTCATGGTTGGTATGGGTC-3′ and 5′-CCTCTGTGAGTAGAACTGGGTGC-3′

*AtJAZ9*: 5′-ATGAGGTTAACGATGATGCTG-3′ and 5′-CTTAGCCTCCTGGAAATCTG-3′

*AtJAZ10*: 5′-GTAGTTTCCGAGATATTCAAGGTG-3′ and 5′-GAACCGAACGAGATTTAGCC-3′

*AtUBQ5*:5′-GACGCTTCATCTCGTCC-3′ and 5′-GTAAACGTAGGTGAGTCC-3′

*AtICS1*: 5′-GGCAGGGAGACTTACG-3′ and 5′-AGGTCCCGCATACATT-3′

### In planta bacterial multiplication assays

*Arabidopsis* plants were planted as previous described [30], [36]. The leaves of five-week old plants were dipped into the bacterial suspensions at an OD_600_ = 0.2 (approximately 2×10^8^ cfu/mL) for 15 seconds. The inoculated plants were then transferred to a growth chamber (22°C and 16/8 light/dark regime, 90% humidity). Bacterial populations were determined as colony forming units (cfu) per cm^2^ using a previously described procedure [50].

### Statistical analysis

Statistical analyses were performed using JMP 8.0 (SAS Institute Inc.).

## Supporting Information

Figure S1 **Subcellular localization of GmJAZ1 in plant cells.** GmJAZ1-YFP was transiently expressed in *N. benthamiana* and the fluorescence was observed at 48 hours post *Agro*-infiltration. DAPI was used to stain the nucleus. This experiment was repeated three times with similar results.
(DOC)

Figure S2 **HopZ1a(C216A) localizes in both cytosol and nucleus. **HopZ1a(C216A) and 3×HA-AtJAZ6 were cloned into a T-DNA binary vector pPH4A-GW-Venus (Jian Yao and Sheng Yang He, unpublished) and pJYP003 [58], respectively. HopZ1a(C216A)-YFP and 3×HA-AtJAZ6 were co-expressed in *N. benthamiana* by *Agrobacterium*-mediated transient transformation. Leaf tissues were collected at two days post infiltration and subjected to nuclear protein fractionation by using nuclear protein Extraction Kit (Sigma). Proteins from different fractionations were detected by Western blots. Histone 3 and UDP are marker proteins that can be detected from nuclear and cytosolic fractions, respectively. Anti-GFP, anti-HA, anti-Histone 3 and anti-UDP antibodies were used to verify the expression of HopZ1a(C216A)-YFP, HA-AtJAZ6 or marker proteins. This experiment was repeated twice with similar results.
(DOC)

Figure S3 **HopZ1a-triggered GmJAZ1 degradation in *N. benthamiana* is independent of plant cell death.** (**A**) GFP protein level was not altered when co-expressed with HopZ1a. GFP was under the control of CaMV 35S promoter and co-expressed in *N. benthamiana* with HopZ1a using *Agrobacterium*-mediated transient expression. The abundance of the GFP protein was determined using anti-GFP antibody at 24 hpi. Anti-HA antibody was used to verify the expression of the HopZ1a proteins. The protein gel was stained with Coomassie blue as a loading control. (**B**) AvrRpt2 did not induce GmJAZ1 degradation although it elicits cell death in *N. benthamiana*. GmJAZ1-FLAG and AvrRpt2-HA were transiently expressed in *N. benthamiana* individually. Total proteins were extracted from the infiltrated leaves at 20 hours post *Agro*-infiltration, mixed in equal volume, and incubated at 4°C for six hours. The abundance of GmJAZ1-FLAG was then analyzed by western blots. The bands corresponding to AvrRpt2 were labeled with *. These experiments were repeated twice with similar results.
(DOC)

Figure S4 **Phylogenetic analysis of GmJAZ1 and AtJAZs. **The PhyML tree was generated using full-length protein sequences by Seaview [59].
(DOC)

Figure S5 **HopZ1a strongly acetylates tag-free AtJAZ6. **Tag-free HopZ1a or HopZ1a(C216A), and AtJAZ6 were purified from *E. coli* and subjected to in vitro acetylation assay. This experiment was repeated twice with similar results.
(DOC)

Figure S6 **HopZ1a no longer activates JA signaling in *coi1-1* mutant *Arabidopsis*.** *Arabidopsis coi1-1, zar1-1* mutant plants were inoculated with *Pto*DC3000 or *Pto*DC3118 carrying the empty pUCP18 vector (EV), HopZ1a or HopZ1a(C216A). Relative expressions of *AtJAZ9*, *AtJAZ10* or *AtICS1* were determined by comparing the normalized transcript levels between the infected and the mock-inoculated samples (leaves infiltrated with 10 mM MgSO_4_). *AtUBQ5* was used as the internal standard. (**A**) Transcript abundances of the JA-responsive genes *AtJAZ9* and *AtJAZ10* were determined at 6 hpi. (**B**) Transcript level of *AtICS1* was determined at 9 hpi. Values are means ± standard deviations (as error bars) (n = 3). All experiments were repeated twice with similar results.
(DOC)

Figure S7 **HopZ1a(G2A) facilitates *Pto*DC3118 infection to the same extent as wild-type HopZ1a.** *Pto*DC3118 expressing the empty vector (EV), HopZ1a, HopZ1a(C216A) or HopZ1a(G2A) were used to dip-inoculate five-week old *Arabidopsis zar1-1* plants. Colony forming units (cfu) were determined at 0 day and 3 dpi. The average colony forming units per square centimeter (cfu/cm^2^) of four biological replicates are presented with error bars showing the standard deviations. Different letters at the top of the bars represent data with statistically significant differences (two tailed t-test *p*<0.01). This experiment was repeated twice with similar results.
(DOC)

Figure S8 **HopZ1a expression in *Pto*DC3000 or *Pto*DC3118 cells was demonstrated by western blots to confirm that differences in the cellular function of the wild type and the catalytic mutant of HopZ1a in planta were not due to different protein expression in *P. syringae*. **Bacterial cells were induced in M63 minimal medium containing 1% fructose. Total proteins from equal amount of the induced cells were extracted and HopZ1a, HopZ1a(C216A) or HopZ1a(G2A) proteins were detected by western blots using anti-HA antibody. These experiments were repeated twice with similar results.
(DOC)

Table S1 **Bacterial strains and plasmids used in this study.**
(PDF)
