# Supplementary material for: Bacterial Effector Activates Jasmonate Signaling by Directly Targeting JAZ Transcriptional Repressors
Source: PLoS Pathog. 2013 Oct 31;9(10):e1003715. doi: 10.1371/journal.ppat.1003715 (PMC3814404; doi:10.1371/journal.ppat.1003715)
Supplement: Figure S1 — Subcellular localization of GmJAZ1 in plant cells. GmJAZ1-YFP was transiently expressed in N. benthamiana and the fluorescence was observed at 48 hours post Agro-infiltration. DAPI was used to stain the nucleus. This experiment was repeated three times with similar results. (DOC) [file ppat.1003715.s001.doc]

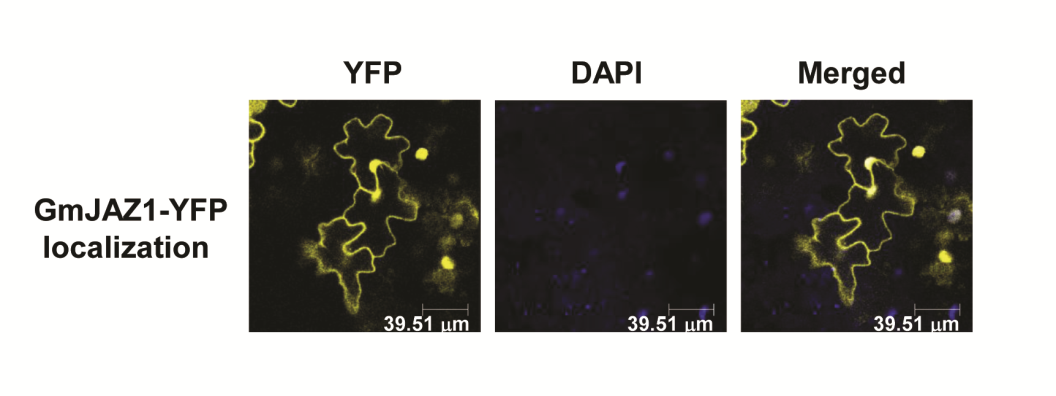


**Figure S1.** Subcellular localization of GmJAZ1 in plant cells. GmJAZ1-YFP was transiently expressed in *N. benthamiana* and the fluorescence was observed at 48 hours post *Agro*-infiltration. DAPI was used to stain the nucleus. This experiment was repeated three times with similar results.
