# Supplementary material for: Bacterial Effector Activates Jasmonate Signaling by Directly Targeting JAZ Transcriptional Repressors
Source: PLoS Pathog. 2013 Oct 31;9(10):e1003715. doi: 10.1371/journal.ppat.1003715 (PMC3814404; doi:10.1371/journal.ppat.1003715)
Supplement: Figure S2 — HopZ1a(C216A) localizes in both cytosol and nucleus. HopZ1a(C216A) and 3×HA-AtJAZ6 were cloned into a T-DNA binary vector pPH4A-GW-Venus (Jian Yao and Sheng Yang He, unpublished) and pJYP003 [58], respectively. HopZ1a(C216A)-YFP and 3×HA-AtJAZ6 were co-expressed in N. benthamiana by Agrobacterium-mediated transient transformation. Leaf tissues were collected at two days post infiltration and subjected to nuclear protein fractionation by using nuclear protein Extraction Kit (Sigma). Proteins from different fractionations were detected by Western blots. Histone 3 and UDP are marker proteins that can be detected from nuclear and cytosolic fractions, respectively. Anti-GFP, anti-HA, anti-Histone 3 and anti-UDP antibodies were used to verify the expression of HopZ1a(C216A)-YFP, HA-AtJAZ6 or marker proteins. This experiment was repeated twice with similar results. (DOC) [file ppat.1003715.s002.doc]

**Figure S2.** HopZ1a(C216A) localizes in both cytosol and nucleus. HopZ1a(C216A) and 3×HA-AtJAZ6 were cloned into a T-DNA binary vector pPH4A-GW-Venus (Jian Yao and Sheng Yang He, unpublished) and pJYP003, respectively. HopZ1a(C216A)-YFP and 3×HA-AtJAZ6 were co-expressed in *N. benthamiana* by *Agrobacterium*-mediated transient transformation. Leaf tissues were collected at two days post infiltration and subjected to nuclear protein fractionation by using nuclear protein Extraction Kit (Sigma). Proteins from different fractionations were detected by Western blots. Histone 3 and UDP are marker proteins that can be detected from nuclear and cytosolic fractions, respectively. Anti-GFP, anti-HA, anti-Histone 3 and anti-UDP antibodies were used to verify the expression of HopZ1a(C216A)-YFP, HA-AtJAZ6 or marker proteins. This experiment was repeated twice with similar results.

REFERENCES:

1. Yang DL, Yao J, Mei CS, Tong XH, Zeng LJ, et al. (2012) Plant hormone jasmonate prioritizes defense over growth by interfering with gibberellin signaling cascade. Proc Natl Acad Sci U S A 109: E1192-1200.
