# Supplementary material for: Bacterial Effector Activates Jasmonate Signaling by Directly Targeting JAZ Transcriptional Repressors
Source: PLoS Pathog. 2013 Oct 31;9(10):e1003715. doi: 10.1371/journal.ppat.1003715 (PMC3814404; doi:10.1371/journal.ppat.1003715)
Supplement: Figure S3 — HopZ1a-triggered GmJAZ1 degradation in N. benthamiana is independent of plant cell death. (A) GFP protein level was not altered when co-expressed with HopZ1a. GFP was under the control of CaMV 35S promoter and co-expressed in N. benthamiana with HopZ1a using Agrobacterium-mediated transient expression. The abundance of the GFP protein was determined using anti-GFP antibody at 24 hpi. Anti-HA antibody was used to verify the expression of the HopZ1a proteins. The protein gel was stained with Coomassie blue as a loading control. (B) AvrRpt2 did not induce GmJAZ1 degradation although it elicits cell death in N. benthamiana. GmJAZ1-FLAG and AvrRpt2-HA were transiently expressed in N. benthamiana individually. Total proteins were extracted from the infiltrated leaves at 20 hours post Agro-infiltration, mixed in equal volume, and incubated at 4°C for six hours. The abundance of GmJAZ1-FLAG was then analyzed by western blots. The bands corresponding to AvrRpt2 were labeled with *. These experiments were repeated twice with similar results. (DOC) [file ppat.1003715.s003.doc]

**A**


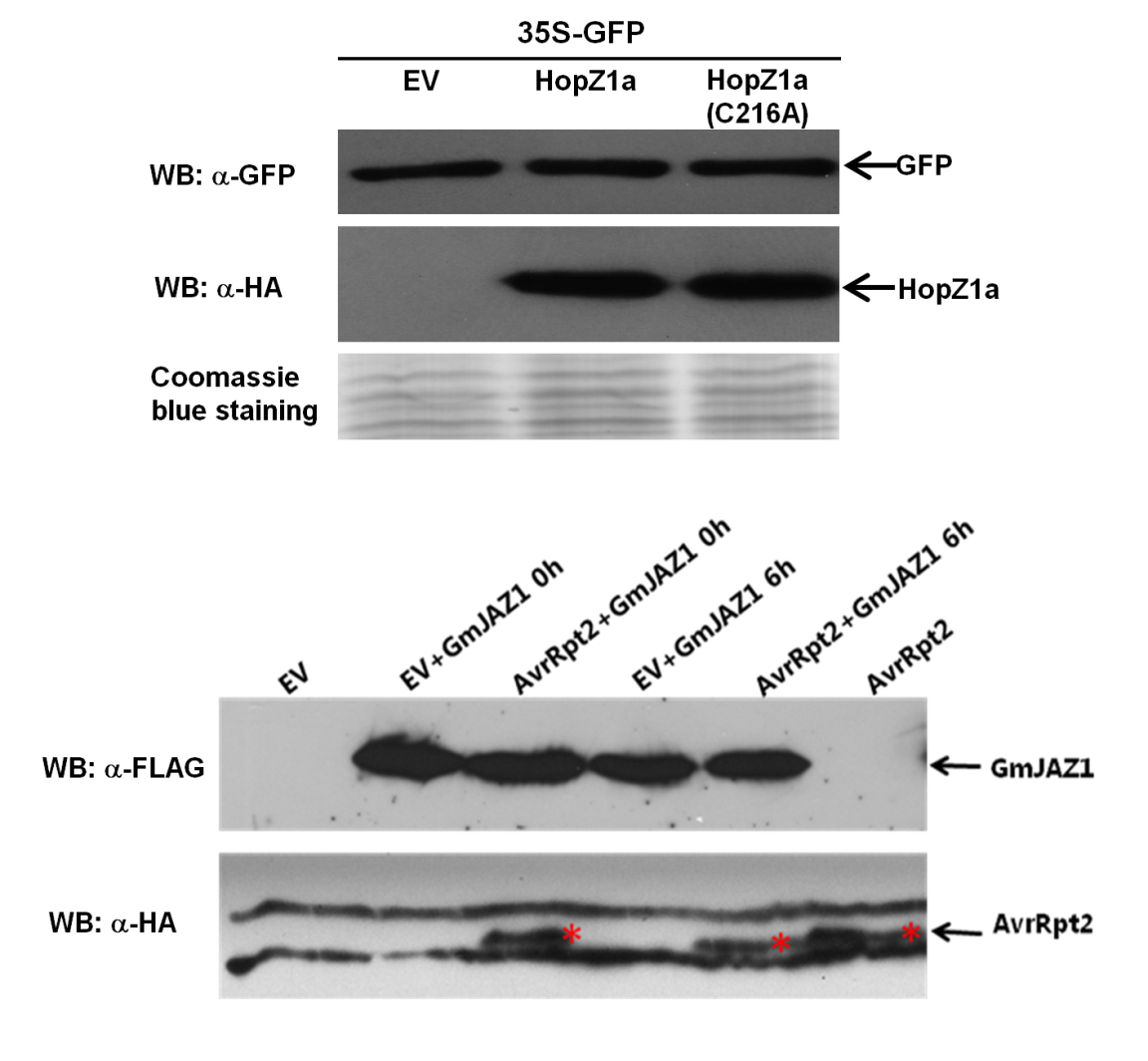


**B**

**Figure S3.** HopZ1a-triggered GmJAZ1 degradation in *N. benthamiana* is independent of plant cell death.

(A) GFP protein level was not altered when co-expressed with HopZ1a. GFP was under the control of CaMV 35S promoter and co-expressed in *N. benthamiana* with HopZ1a using *Agrobacterium*-mediated transient expression. The abundance of the GFP protein was determined using anti-GFP antibody at 24 hpi. Anti-HA antibody was used to verify the expression of the HopZ1a proteins. The protein gel was stained with Coomassie blue as a loading control.

(B) AvrRpt2 did not induce GmJAZ1 degradation although it elicits cell death in *N. benthamiana*. GmJAZ1-FLAG and AvrRpt2-HA were transiently expressed in *N. benthamiana* individually. Total proteins were extracted from the infiltrated leaves at 20 hours post *Agro*-infiltration, mixed in equal volume, and incubated at 4°C for six hours. The abundance of GmJAZ1-FLAG was then analyzed by western blots. The bands corresponding to AvrRpt2 were labeled with *.

These experiments were repeated twice with similar results.
