# Supplementary material for: Bacterial Effector Activates Jasmonate Signaling by Directly Targeting JAZ Transcriptional Repressors
Source: PLoS Pathog. 2013 Oct 31;9(10):e1003715. doi: 10.1371/journal.ppat.1003715 (PMC3814404; doi:10.1371/journal.ppat.1003715)
Supplement: Figure S4 — Phylogenetic analysis of GmJAZ1 and AtJAZs. The PhyML tree was generated using full-length protein sequences by Seaview [59]. (DOC) [file ppat.1003715.s004.doc]

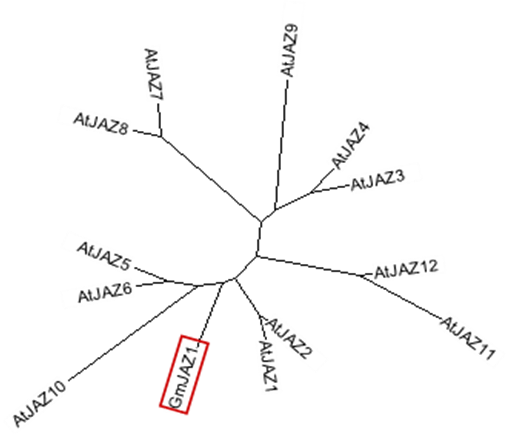


**Figure S4.** Phylogenetic analysis of GmJAZ1 and AtJAZs. The PhyML tree was generated using full-length protein sequences by Seaview.

REFERENCES:

1. Gouy M, Guindon S, Gascuel O (2010) SeaView version 4: A multiplatform graphical user interface for sequence alignment and phylogenetic tree building. Mol Biol Evol 27: 221-224.
