# Supplementary material for: Bacterial Effector Activates Jasmonate Signaling by Directly Targeting JAZ Transcriptional Repressors
Source: PLoS Pathog. 2013 Oct 31;9(10):e1003715. doi: 10.1371/journal.ppat.1003715 (PMC3814404; doi:10.1371/journal.ppat.1003715)
Supplement: Figure S5 — HopZ1a strongly acetylates tag-free AtJAZ6. Tag-free HopZ1a or HopZ1a(C216A), and AtJAZ6 were purified from E. coli and subjected to in vitro acetylation assay. This experiment was repeated twice with similar results. (DOC) [file ppat.1003715.s005.doc]

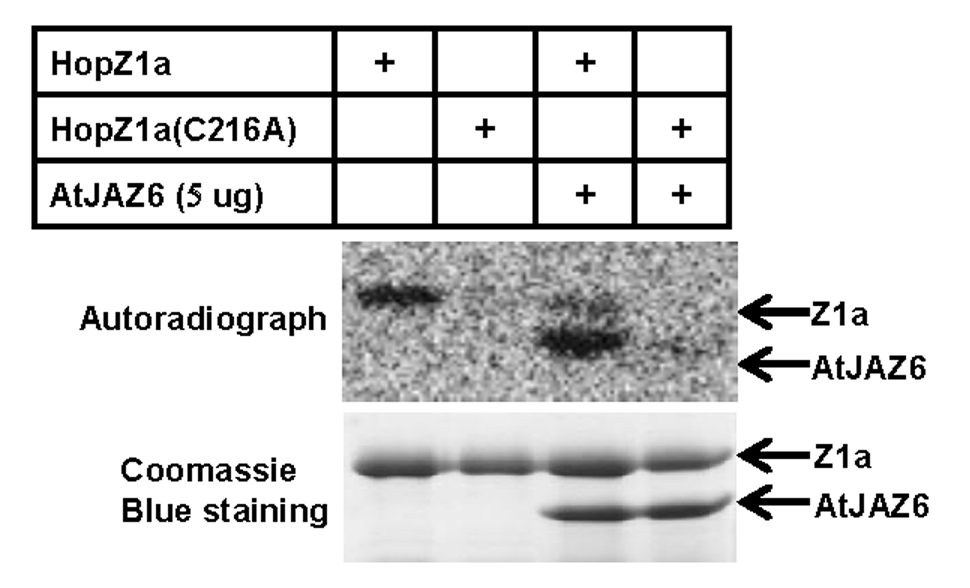


**Figure S5.** HopZ1a strongly acetylates tag-free AtJAZ6. Tag-free HopZ1a or HopZ1a(C216A), and AtJAZ6 were purified from *E. coli* and subjected to in vitro acetylation assay. This experiment was repeated twice with similar results.
