# Supplementary material for: Bacterial Effector Activates Jasmonate Signaling by Directly Targeting JAZ Transcriptional Repressors
Source: PLoS Pathog. 2013 Oct 31;9(10):e1003715. doi: 10.1371/journal.ppat.1003715 (PMC3814404; doi:10.1371/journal.ppat.1003715)
Supplement: Figure S6 — HopZ1a no longer activates JA signaling in coi1-1 mutant Arabidopsis. Arabidopsis coi1-1, zar1-1 mutant plants were inoculated with PtoDC3000 or PtoDC3118 carrying the empty pUCP18 vector (EV), HopZ1a or HopZ1a(C216A). Relative expressions of AtJAZ9, AtJAZ10 or AtICS1 were determined by comparing the normalized transcript levels between the infected and the mock-inoculated samples (leaves infiltrated with 10 mM MgSO4). AtUBQ5 was used as the internal standard. (A) Transcript abundances of the JA-responsive genes AtJAZ9 and AtJAZ10 were determined at 6 hpi. (B) Transcript level of AtICS1 was determined at 9 hpi. Values are means ± standard deviations (as error bars) (n = 3). All experiments were repeated twice with similar results. (DOC) [file ppat.1003715.s006.doc]

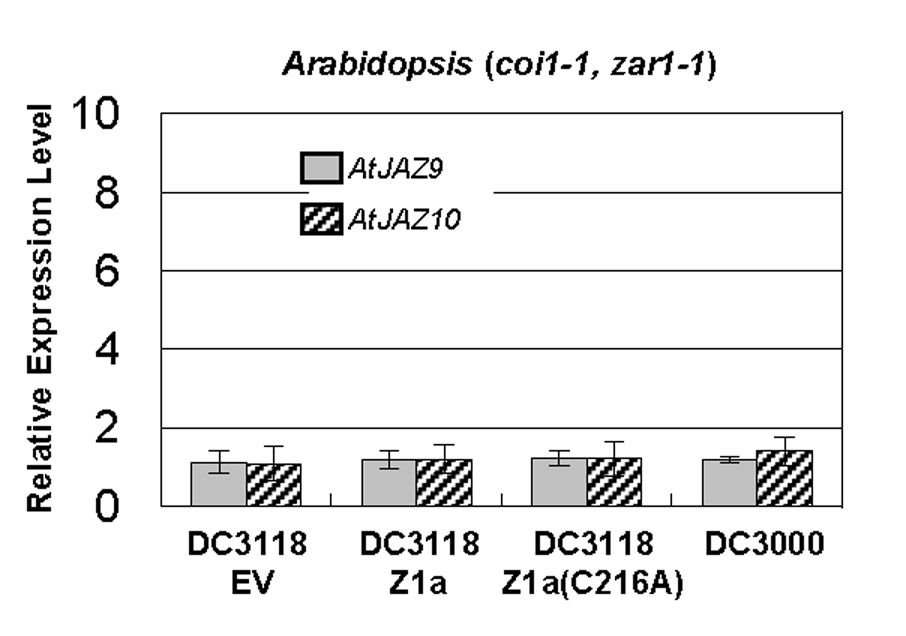


**A**


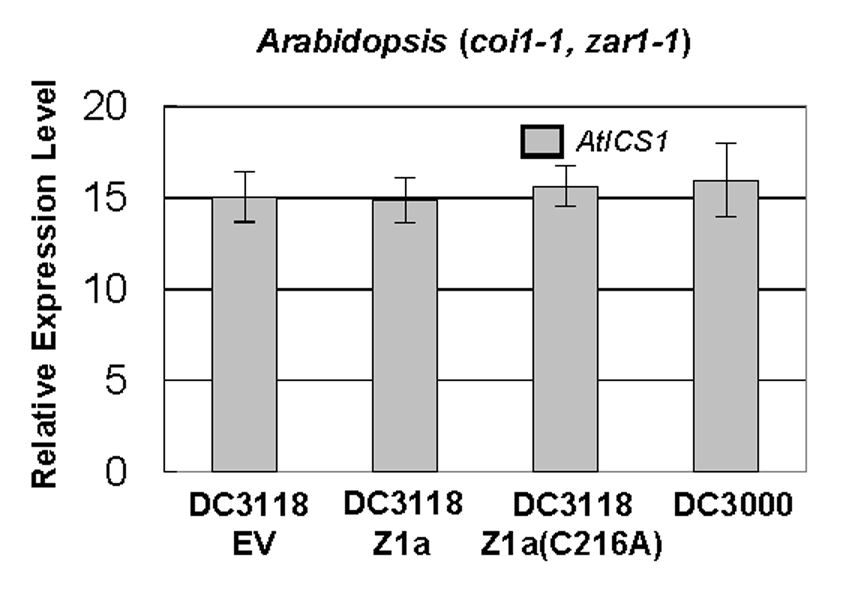


**B**

**Figure S6.** HopZ1a no longer activates JA signaling in *coi1-1* mutant *Arabidopsis*. *Arabidopsis coi1-1, zar1-1* mutant plants were inoculated with *Pto*DC3000 or *Pto*DC3118 carrying the empty pUCP18 vector (EV), HopZ1a or HopZ1a(C216A). Relative expression was determined by comparing the normalized *AtJAZ9*, *AtJAZ10* or *AtICS1* transcript levels between the infected and the mock-inoculated samples (leaves infiltrated with 10mM MgSO_4_). *AtUBQ5* was used as the internal standard.

(A) Transcript abundances of the JA-responsive genes *AtJAZ9* and *AtJAZ10* were determined at 6 hpi.

(B) Transcript level of *AtICS1* was determined at 9 hpi. Values are means + standard deviations (as error bars) (n=3).

All experiments were repeated twice with similar results.
