# Supplementary material for: Bacterial Effector Activates Jasmonate Signaling by Directly Targeting JAZ Transcriptional Repressors
Source: PLoS Pathog. 2013 Oct 31;9(10):e1003715. doi: 10.1371/journal.ppat.1003715 (PMC3814404; doi:10.1371/journal.ppat.1003715)
Supplement: Figure S7 — HopZ1a(G2A) facilitates PtoDC3118 infection to the same extent as wild-type HopZ1a. PtoDC3118 expressing the empty vector (EV), HopZ1a, HopZ1a(C216A) or HopZ1a(G2A) were used to dip-inoculate five-week old Arabidopsis zar1-1 plants. Colony forming units (cfu) were determined at 0 day and 3 dpi. The average colony forming units per square centimeter (cfu/cm2) of four biological replicates are presented with error bars showing the standard deviations. Different letters at the top of the bars represent data with statistically significant differences (two tailed t-test p<0.01). This experiment was repeated twice with similar results. (DOC) [file ppat.1003715.s007.doc]

**
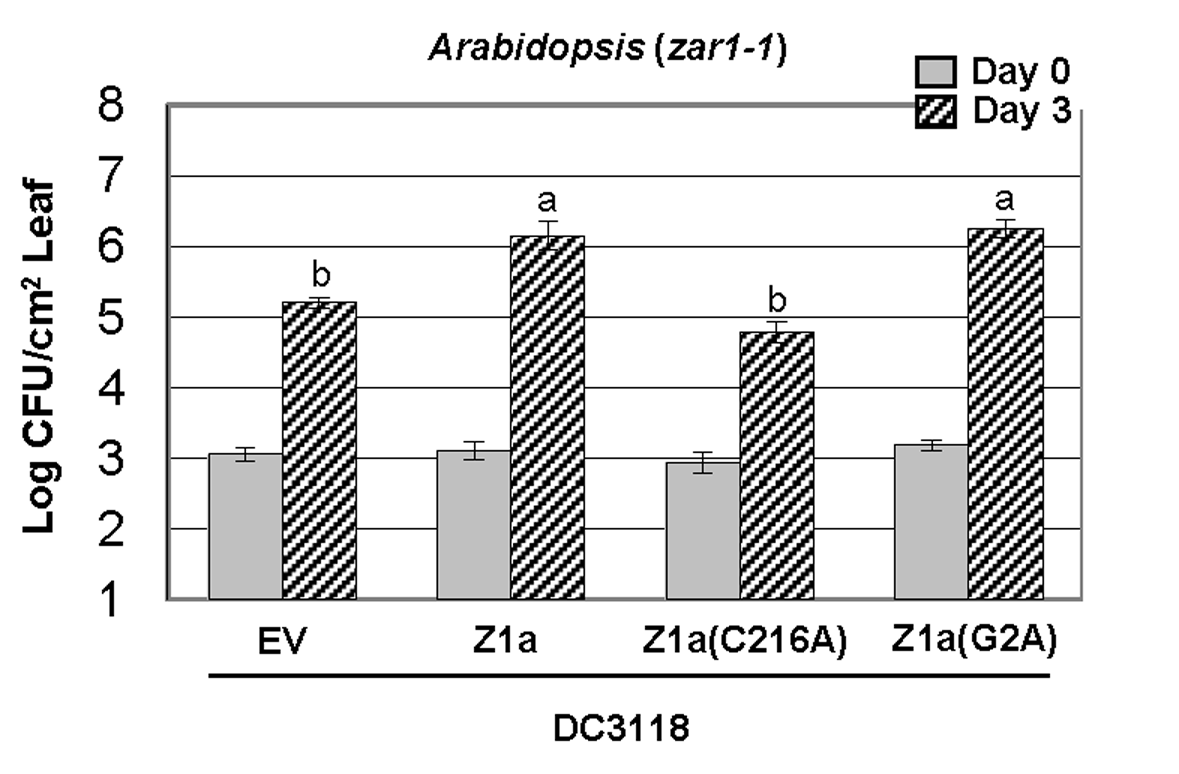
**

**Figure S7.** HopZ1a(G2A) facilitates *Pto*DC3118 infection to the same extent as wild-type HopZ1a. *Pto*DC3118 expressing the empty vector (EV), HopZ1a, HopZ1a(C216A) or HopZ1a(G2A) were used to dip-inoculate five-week old *Arabidopsis* *zar1-1* plants. Colony forming units (cfu) were determined at 0 day and 3 dpi. The average colony forming units per square centimeter (cfu/cm^2^) of four biological replicates are presented with error bars showing the standard deviations. Different letters at the top of the bars represent data with statistically significant differences (two tailed t-test *p*<0.01). This experiment was repeated twice with similar results.
