# Supplementary material for: Bacterial Effector Activates Jasmonate Signaling by Directly Targeting JAZ Transcriptional Repressors
Source: PLoS Pathog. 2013 Oct 31;9(10):e1003715. doi: 10.1371/journal.ppat.1003715 (PMC3814404; doi:10.1371/journal.ppat.1003715)
Supplement: Figure S8 — HopZ1a expression in PtoDC3000 or PtoDC3118 cells was demonstrated by western blots to confirm that differences in the cellular function of the wild type and the catalytic mutant of HopZ1a in planta were not due to different protein expression in P. syringae. Bacterial cells were induced in M63 minimal medium containing 1% fructose. Total proteins from equal amount of the induced cells were extracted and HopZ1a, HopZ1a(C216A) or HopZ1a(G2A) proteins were detected by western blots using anti-HA antibody. These experiments were repeated twice with similar results. (DOC) [file ppat.1003715.s008.doc]

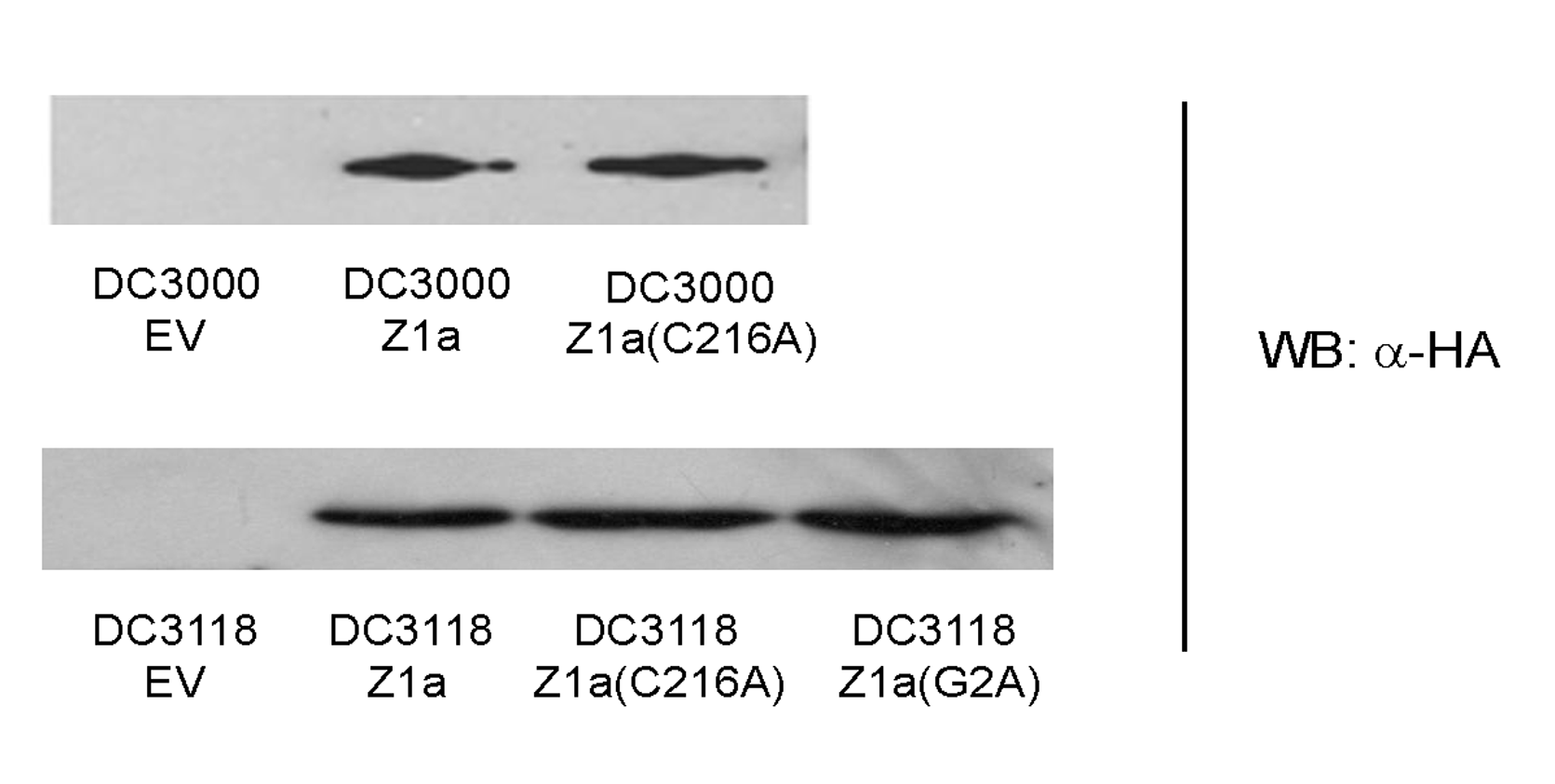


**Figure S8.** HopZ1a expression in *Pto*DC3000 or *Pto*DC3118 cells was demonstrated by western blots to confirm that differences in the cellular function of the wild type and the catalytic mutant of HopZ1a in planta were not due to different protein expression in *P. syringae*. Bacterial cells were induced in M63 minimal medium containing 1% fructose. Total proteins from equal amount of the induced cells were extracted and HopZ1a, HopZ1a(C216A) or HopZ1a(G2A) proteins were detected by western blots using anti-HA antibody. These experiments were repeated twice with similar results.
