# Supplementary material for: Bacterial Effector Activates Jasmonate Signaling by Directly Targeting JAZ Transcriptional Repressors
Source: PLoS Pathog. 2013 Oct 31;9(10):e1003715. doi: 10.1371/journal.ppat.1003715 (PMC3814404; doi:10.1371/journal.ppat.1003715)
Supplement: Table S1 — Bacterial strains and plasmids used in this study. (PDF) [file ppat.1003715.s009.pdf]

Table S1. Bacterial strains and plasmids used in this study

Strains or Plasmids	Description	Source/ reference
Escherichia coli DH5α	F- Φ80d*lac*ZΔM15 Δ(*lac*ZYA-*arg*F) U169 *rec*A1 *end*A1, *hsd*R17(rk-, mk+) *pho*A *sup*E44 λ- *thi*-1 *gyr*A96 *rel*A1	Invitrogen
Escherichia coli BL21(DE3)	F^- *ompT gal dcm lon* $hsdS_B(r_B^- m_B^-)$ λ(DE3 [*lacI lacUV5-T7 gene 1 ind1 sam7 nin5*]	Invitrogen
Pseudomonas syringae pv. tomato strain DC3000	Isolated from tomato plants, also infects *Arabidopsis*, Rif^R	Cuppels, 1986
Pseudomonas syringae pv. tomato strain DC3118	A mutant of *Pto*DC3000 that does not produce coronatine, Rif^R, Kan^R	Melloto et al., 2006; Moore et al, 1989
Agrobacterium tumefaciens GV3101(pMP90)	Rif^R, $Gent^R$	Holsters, 1980
Agrobacterium tumefaciens C58C1 (pCH32)	Rif^R, Tet^R	Mudgett et al., 2000
pUCP18	Plasmid vector multiplies in *P. syringae,* Amp^R	Schweizer, 1991
pUCP18::*HopZ1a-HA*	pUCP18 carrying the gene encoding HopZ1a tagged with HA and under the control of the native promoter, Amp^R	This study
pUCP18::*HopZ1a(C216A)-HA*	pUCP18 carrying the gene encoding the HopZ1a catalytic mutant with Cys216 replaced with an alanine, Amp^R	This study
pUCP18::*HopZ1a(G2A)-HA*	pUCP18 carrying the gene encoding the HopZ1a mutant with Gly2 replaced with an alanine, Amp^R	This study
pDSK600::*avrRpt2*	pDSK600 carrying *avrRpt2* gene under the control of its own promoter, Rif^R, Kan^R	Mudgett and Staskawicz, 1999
pUCP20tk	Plasmid vector multiplies in *P. syringae*, Kan^R	Zhou et al., 2009
pUCP20tk::*hopZ1a-HA*	pUCP20tk carrying the gene encoding HopZ1a tagged with HA and under the control of the native promoter, Kan^R	Ma et al., 2006
pUCP20tk::*hopZ1a(C216A)-HA*	pUCP20tk carrying the gene encoding the HopZ1a catalytic mutant with Cys216 replaced with an alanine, Kan^R	Zhou et al., 2009
pMDD1	A binary vector with cauliflower mosaic virus *35S* promoter, Kan^R	Mudgett et al., 2000
pMDD1::*hopZ1a-HA*	pMDD1 carrying *hopZ1a* tagged with HA, Kan^R	Zhou et al., 2009
pMDD1::*hopZ1a(C216A)-HA*	pMDD1 carrying *hopZ1a(C216A)* tagged with HA, Kan^R	Zhou et al., 2009
pMD1::*avrRpt2-HA*	pMD1 carrying *avrRpt2* tagged with HA, Kan^R	G. Coaker

pEG100	pEarleyGate100, a Gateway binary vector with cauliflower mosaic virus *35S* promoter, Kan^R	Earley et al., 2006
pEG100::*GmJAZ1-FLAG*	pEG100 carrying *GmJAZ1* tagged with FLAG at the C-terminus, Kan^R	This study
pEG100::*3×FLAG-HopZ1a*	pEG100 carrying *hopZ1a* tagged with 3×FLAG at N-terminus, Kan^R	This study
pEG100::*3×FLAG-HopZ1a(C216A)*	pEG100 carrying *hopZ1a(C216A)* tagged with 3×FLAG at N-terminus, Kan^R	This study
pEG101	pEarleyGate101, a Gateway binary vector for YFP fusion protein expression with cauliflower mosaic virus *35S* promoter, Kan^R	Earley et al., 2006
pEG101::*GmJAZ1*	*GmJAZ1* is in-frame fused to YFP and HA, Kan^R	This study
pEG101::*AtJAZ6*	*AtJAZ6* is in-frame fused to YFP and HA, Kan^R	This study
pEG101::*AtJAZ6ΔJas*	*AtJAZ6ΔJas* (with 10 aa deletion in the Jas domain) is in-frame fused to YFP and HA, Kan^R	This study
pSPYNE	A binary vector with cauliflower mosaic virus *35S* promoter and the N-terminal (1-155 aa) domain of YFP (nYFP), Kan^R	Walter et al., 2004
pSPYCE	A binary vector with cauliflower mosaic virus 35s promoter and the C-terminal (156-239 aa) domain of YFP (cYFP), Kan^R	Walter et al., 2004
pSPYNE::*hopZ1a(C216A)*	pSPYNE carrying *hopZ1a(C216A)* in-frame fused with nYFP, Kan^R	Zhou et al., 2011
pSPYCE::*GmJAZ1*	pSPYCE carrying *GmJAZ1* in-frame fused with cYFP, Kan^R	This study
pSPYCE::*AtJAZ6*	pSPYCE carrying *AtJAZ6* in-frame fused with cYFP, Kan^R	This study
pGEX4T-2	*E. coli* expression vector with an N-terminal GST tag, Amp^R	Amersham
pGEX4T-2::*hopZ1a*	pGEX4T-2 carrying *hopZ1a*, Amp^R	Zhou et al., 2011
pGEX4T-2::*hopZ1a(C216A)*	pGEX4T-2 carrying *hopZ1a(C216A)*, Amp^R	This study
pET14b	*E. coli* expression vector with an N-terminal 6×His tag, Amp^R	Novagen
pET14b::*GmJAZ1*	pET14b carrying *GmJAZ1* with an N-terminal 6×His tag, Amp^R	This study
pET-mal	*malE* gene from pMAL-c2 is cloned into *Nde*I-*Xho*I site of pET28a, Kan^R	Sweeney et al., 2005
pET-mal::*AtJAZ*	pET-mal carrying *AtJAZ* genes, Kan^R	This study
pET-mal::*AtJAZ6ΔJas*	pET-mal carrying *AtJAZ6ΔJas* (with 10 aa deletion in the Jas domain), Kan^R	This study
pENTR/D-TOPO	An entry vector for the Gateway system, Kan^R	Invitrogen
pENTR/D-TOPO::*3×FLAG-hopZ1a*	pENTR/D-TOPO carrying the *hopZ1a* gene with a 3×FLAG tag at the N-terminus, Kan^R	This study
pENTR/D-TOPO::*3×FLAG-hopZ1a(C216A)*	pENTR/D-TOPO carrying the *hopZ1a(C216A)* gene with a 3×FLAG tag at the N-terminus, Kan^R	This study

pPH4A-GW-Venus::*HopZ1a(C216A)*	T-DNA binary vector pPH4A-GW-Venus carrying *HopZ1a(C216A)*, $Spec^R$	This study
pJYP003::*3×HA-AtJAZ6*	pJYP003 carrying *AtJAZ6* with a 3×HA tag at the N-terminus, Kan^R	Yang et al., 2012

References

1. Cuppels DA (1986) Generation and characterization of Tn5 insertion mutations in Pseudomonas syringae pv. tomato. *Appl. Environ. Microbiol.* 51: 323-327.
2. Earley KW, Haag JR, Pontes O, Opper K, Juehne T, et al. (2006) Gateway-compatible vectors for plant functional genomics and proteomics. *Plant J* 45: 616-629.
3. Holsters M, Silva B, Van Vlient F, Genetello C, De Block M, et al. (1980) The functional organization of the nopaline *A. tumefaciens* plasmid pTiC58. *Plasmid* 3: 212–230.
4. Ma W, Dong FF, Stavrinides J, Guttman DS (2006) Type III effector diversification via both pathoadaptation and horizontal transfer in response to a coevolutionary arms race. PLoS Genet 2: e209.
5. Melotto M, Underwood W, Koczan J, Nomura K, He SY (2006) Plant stomata function in innate immunity against bacterial invasion. *Cell* 126**:** 969-980.
6. Moore RA, Starratt AN, Ma SW, Morris VL, Cuppels DA (1989) Identification of a Chromosomal Region Required for Biosynthesis of the Phytotoxin Coronatine by Pseudomonas-Syringae Pv Tomato. Canadian Journal of Microbiology 35: 910-917.
7. Mudgett MB, Chesnokova O, Dahlbeck D, Clark ET, Rossier O, et al. (2000) Molecular signals required for type III secretion and translocation of the Xanthomonas campestris AvrBs2 protein to pepper plants. *Proc Natl Acad Sci U S A* 97: 13324-13329.
8. Mudgett MB and Staskawicz BJ (1999) Characterization of the Pseudomonas syringae pv. tomato AvrRpt2 protein: demonstration of secretion and processing during bacterial pathogenesis. Molecular Microbiology 32(5): 927-941.
9. Schweizer HP (1991) Escherichia-Pseudomonas shuttle vectors derived from pUC18/19. *Gene* 97: 109-112.
10. Sweeney MC, Wavreille AS, Park J, Butchar JP, Tridandapani S, et al. (2005) Decoding protein-protein interactions through combinatorial chemistry: sequence specificity of SHP-1, SHP-2, and SHIP SH2 domains. *Biochemistry* 44**:** 14932-14947.
11. Walter M, Chaban C, Schutze K, Batistic O, Weckermann K, et al. (2004) Visualization of protein interactions in living plant cells using bimolecular fluorescence complementation. *Plant J* 40**:** 428-438.
12. Yang DL, Yao J, Mei CS, Tong XH, Zeng LJ, et al.(2012) Plant hormone jasmonate prioritizes defense over growth by interfering with gibberellin signaling cascade. Proceedings of the National Academy of Sciences of the United States of America 109: E1192-E1200.
13. Zhou H, Morgan RL, Guttman DS, Ma W (2009) Allelic variants of the Pseudomonas syringae type III effector HopZ1 are differentially recognized by plant resistance systems. *Mol Plant Microbe Interact* 22**:** 176-189.

14. Zhou H, Lin J, Johnson A, Morgan RL, Zhong W, et al. (2011) Pseudomonas syringae type III effector HopZ1 targets a host enzyme to suppress isoflavone biosynthesis and promote infection in soybean. *Cell Host Microbe* 9**:** 177-186.
